# Structural Determinants for Activity and Specificity of the Bacterial Toxin LlpA

**DOI:** 10.1371/journal.ppat.1003199

**Published:** 2013-02-28

**Authors:** Maarten G. K. Ghequire, Abel Garcia-Pino, Eline K. M. Lebbe, Stijn Spaepen, Remy Loris, René De Mot

**Affiliations:** 1 Centre of Microbial and Plant Genetics, University of Leuven, Heverlee-Leuven, Belgium; 2 Molecular Recognition Unit, Department of Structural Biology, Vlaams Instituut voor Biotechnologie, Brussel, Belgium; 3 Structural Biology Brussels, Department of Biotechnology (DBIT), Vrije Universiteit Brussel, Brussel, Belgium; Geisel School of Medicine at Dartmouth, United States of America

## Abstract

Lectin-like bacteriotoxic proteins, identified in several plant-associated bacteria, are able to selectively kill closely related species, including several phytopathogens, such as *Pseudomonas syringae* and *Xanthomonas* species, but so far their mode of action remains unrevealed. The crystal structure of LlpA_BW_, the prototype lectin-like bacteriocin from *Pseudomonas putida*, reveals an architecture of two monocot mannose-binding lectin (MMBL) domains and a C-terminal β-hairpin extension. The C-terminal MMBL domain (C-domain) adopts a fold very similar to MMBL domains from plant lectins and contains a binding site for mannose and oligomannosides. Mutational analysis indicates that an intact sugar-binding pocket in this domain is crucial for bactericidal activity. The N-terminal MMBL domain (N-domain) adopts the same fold but is structurally more divergent and lacks a functional mannose-binding site. Differential activity of engineered N/C-domain chimers derived from two LlpA homologues with different killing spectra, disclosed that the N-domain determines target specificity. Apparently this bacteriocin is assembled from two structurally similar domains that evolved separately towards dedicated functions in target recognition and bacteriotoxicity.

## Author Summary

In their natural environments, microorganisms compete for space and nutrients, and a major strategy to assist in niche colonization is the deployment of antagonistic compounds directed at competitors, such as secondary metabolites (antibiotics) and antibacterial peptides or proteins (bacteriocins). The latter selectively kill closely related bacteria, which is also the case for members of the LlpA family. Here, we investigate the structure-function relationship for the prototype LlpA_BW_ from a saprophytic plant-associated *Pseudomonas* whose genus-specific target spectrum includes several phytopathogenic pseudomonads. By determining the 3D structure of this protein, we could assign LlpA to the so-called monocot mannose-binding lectin (MMBL) family, representing its first prokaryotic member, and also add a new type of protective function, as the eukaryotic MMBL members have been linked with antiviral, antifungal, nematicidal or insecticidal activities. For the protein containing two similarly folded domains, we constructed site-specific mutants affected in carbohydrate binding and domain chimers from LlpA homologues to show that mannose-specific sugar binding mediated by one domain is required for activity and that the other domain determines target strain specificity. The strategy that evolved for these bacteriocins is reminiscent of the one used by mammalian bactericidal proteins of the RegIII family that recruited a C-type lectin fold to kill bacteria.

## Introduction

In most natural settings, complex interactions occur among microorganisms, ranging from nutritional co-operation to warfare among competitors. Examples of such interplay have been reported not only between unrelated microorganisms (e.g. fungi and bacteria [1], [2]), but also between distant relatives (e.g. members of different bacterial genera [3]), and even between close relatives (e.g. at inter- and intra-species levels [4], [5]). A major strategy in niche colonization is the production of growth inhibitors or toxins directed at microbial competitors [6]. While a huge variety of secondary metabolites is used to target phylogenetically-distant competitors, ribosome-synthesized peptides or proteins are typically active against close relatives. These protein toxins are collectively referred to as bacteriocins, and may either be released into the environment or transferred to the host via specialized contact-dependent delivery systems [7]–[9].

Bacteriocins are structurally and mechanistically very diverse. This is reflected in the bacteriocinogenic potential of the genus *Pseudomonas* [10]. Their R- and F-type pyocins are multi-subunit protein complexes evolutionarily related to contractile tails of bacteriophages [11]–[13]. R-pyocins attach to specific lipopolysaccharide moieties at the cell surface of susceptible cells and insert their core structure through the cell envelope, causing depolarization of the cytoplasmic membrane [14]. The S-type pyocins of *Pseudomonas aeruginosa* share structural and functional features with *Escherichia coli* colicins [15]. Following docking onto surface-exposed targets such as siderophore receptors [16], [17], S-pyocins kill cells by nucleic acid degradation [10], [17], cytoplasmic membrane damage [18], or inhibition of peptidoglycan synthesis [19], [20]. Putidacin A (or LlpA_BW_), first identified in *Pseudomonas putida* BW11M1 [21], represents a class of *Pseudomonas*-specific antibacterial proteins not related to any known bacteriocin. Additional *llpA*-like genes encoding functional bacteriocins were identified by genome mining in the biocontrol strain *Pseudomonas fluorescens* Pf-5 [22] and in the phytopathogen *Pseudomonas syringae* pv. *syringae* 642 [23]. Identification of this type of protein in two *Xanthomonas* pathovars extended its occurrence as a genus-specific killer protein [23]. The *Xanthomonas* LlpA precursor is proteolytically processed by removal of a characteristic Type II secretion signal peptide, whereas such N-terminal sequence is lacking in *Pseudomonas* homologues, indicating that secretory routes may differ among LlpA producers.

The amino acid sequence of LlpA suggests the presence of two related domains belonging to the ‘monocot mannose-binding lectin’ (MMBL) family [24]. The MMBL domain consists of a β-prism fold containing three potential carbohydrate-binding pockets, each generated by a QxDxNxVxY sequence (with x, any amino acid), but some sites may be inactive due to degeneracy of the signature motif [25]. This domain (Pfam domain: B_lectin - PF01453) was initially identified in lectins of monocot plants [26], [27], but a more widespread occurrence of MMBL lectins has become evident and includes representatives in fungi [28], [29], slime molds [30], sponges [31], and fishes [32]–[34]. The LlpA branch occupies a unique position among MMBL-domain proteins, harboring non-eukaryotic representatives and being equipped with the capacity to kill bacterial cells with bacteriocin-like specificity, a property not yet demonstrated for other family members [25]. Next to proteins with the LlpA-type tandem-MMBL organization, many other predicted MMBL proteins are encoded by bacterial genomes. Often the MMBL module is embedded in a larger protein. For one such protein, bacteriocin-like activity among *Ruminococcus* species, Gram-positive bacteria colonizing the rumen, was demonstrated [35].

Here we report on the crystal structure of LlpA_BW_ as the prototype of a novel family of antibacterial proteins and explore how domain architecture and specific structural elements contribute to its activity and specificity.

## Results

### LlpA forms a rigid MMBL tandem

**Figure 1:**
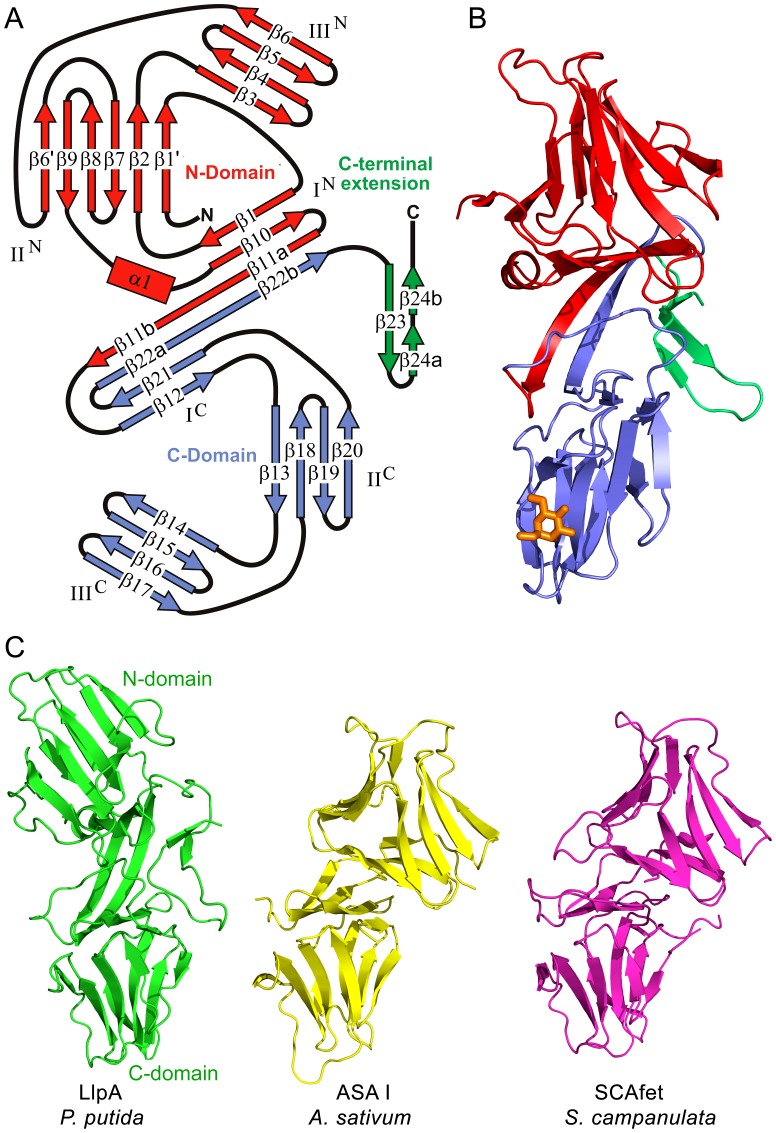
Overall structure of LlpA_BW_. (A) Topology diagram of LlpA_BW_. The N-domain is shown in red, the C-domain in blue and the C-terminal extension in green. The different strands and subdomains are labeled. Domain swapping involves β-strand segments β11b and β22b, which together with β-strand segments β11a and β22a link both MMBL domains. (B) Cartoon representation of LlpA_BW_ with the different domains colored as in panel A. The bound Me-Man residue is shown as an orange stick representation. (C) Domain orientations of LlpA_BW_ compared with the heterodimeric MMBL ASA I (*Allium sativum* agglutinin, PDB entry 1KJ1) and tandem MMBL SCAfet (*Scilla campanulata* fetuin-binding lectin, PDB entry 1DLP). In each case, the C-domain is shown in the same orientation, highlighting the different relative orientation of the N-domain in LlpA_BW_. Domain-swapped dimers in homo-oligomeric plant MMBL lectins such as snowdrop lectin have their domain orientation similar to ASA I and SCAfet.

The crystal structure of LlpA_BW_ from *P. putida* BW11M1 (LlpA_BW_) shows it contains two β-prism MMBL domains, referred to as the N-domain and the C-domain following their position in the amino acid sequence (Figure 1A,B; Figure S1). The N-domain spans residues Arg4-Pro135 while the C-domain encompasses residues Ala136-Gln253. Each domain exhibits pseudo-threefold symmetry and the corresponding subdomains will be referred to as I^N^, II^N^, III^N^, I^C^, II^C^ and III^C^, respectively (Figure 1A and Figure S1). Following these two domains, a β-hairpin extension is formed by residues Pro254-His275 (the numbering used in this paper corresponds to that of the wild-type protein without His-tag [21]).

The two-domain architecture reflects the β-strand swapping that is typical in dimers of single-domain mannose-binding monocot lectins (Figure 1A,B) [36] and which apparently is retained after the ancestral fusion or duplication of the two domains, as is also the case in certain MMBL tandems or heterodimers from monocots [37], [38]. Thus, residues Asp126-Pro135 from the first MMBL sequence complement the fold of the C-domain while residues Pro245-Gln253 from the second MMBL sequence complement the fold of the N-domain. However, in LlpA_BW_, the relative orientation of both domains is different compared to what is observed in a canonical MMBL lectin dimer, such as snowdrop lectin [36], in the heterodimeric MMBL lectin ASA I from *Allium sativum* [38], or in the tandem MMBL SCAfet from *Scilla campanulata* [37] (Figure 1C and Figure S2). In contrast to these plant MMBL proteins, the resulting architecture of LlpA_BW_ does not obey pseudo-twofold symmetry (Figure 1C).

**Figure 2:**
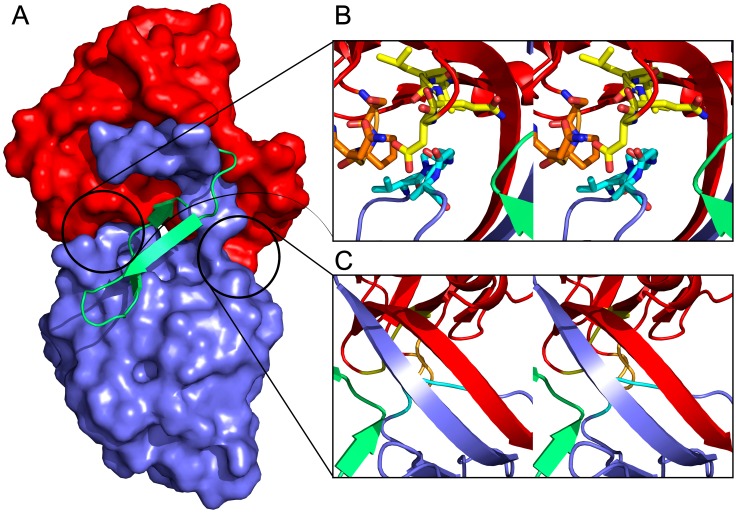
Domain interactions within LlpA_BW_. (A) The C-terminal hairpin extension (green cartoon) covers the interface between the N-domain (red surface representation) and the C-domain (blue surface representation). (B) Stereo view of the interactions between loop segments Val140-Asp145 (cyan) of the C-domain and Val115-Asp118 (yellow) and Ser31-Gln34 (orange) of the N-domain. Other structural elements are colored according to panel A. (C) Stereo view of the two-stranded β-sheet formed by strands β11a,b and β22a,b that links the N- and the C-domains and gives rise to domain swapping. Colors according to panel A and B.

LlpA_BW_ is a very rigid molecule. The two monomers present in the asymmetric unit are essentially identical with a root-mean-square deviation (RMSD) of 0.34 Å for 270 Cα atoms. This RMSD value does not change significantly when the individual domains are fitted separately (0.32 Å for 120 Cα's of the N-domain and 0.22 Å for 115 Cα's of the C-domain), indicating that the inter-domain orientation is fixed. This stems from three sets of interactions (Figure 2). Both domains are connected by a two-stranded anti-parallel β-sheet that is involved in the β-strand swapping mentioned above and that links both domains. The C-terminal β-hairpin extension makes extensive contacts, through hydrophobic and hydrogen bonds, with both domains. Finally, the stretch Val140-Asp145 of the C-domain makes extensive contacts with stretch Val115-Asp118 and with the side chains of Ser15 and Pro32 of the N-domain.

### Domains of LlpA_BW_ are shaped by differential evolutionary pressure

A superposition of the Cα-trace of the N- and C-domain of LlpA_BW_ as well as the MMBL domain of snowdrop lectin is shown in Figure S3. Based on 79 Cα atoms that form the common β-sheet core of the MMBL domains, the RMSD between the N- and C-domains of LlpA_BW_ is 1.84 Å. While the secondary structure elements of the C-domain are restricted to the three four-stranded β-sheets of the β-prism fold, the N-domain contains three additional secondary structure elements (Figure 1A). A three-turn α-helix (α1) is inserted in the loop between strands β9 and β10, and sheet II^N^ contains two additional strands. Strand β6′ is inserted in the loop between strands β6 and β7 and provides an anti-parallel extension to sheet II (hydrogen bonding to strand β9). Strand β1′ is a short piece of β-strand that is part of the long N-terminus and forms a parallel extension on the opposite site of sheet II^N^ (hydrogen bonding to strand β2), making this β-sheet a mixed type six-stranded one rather than the canonical four-stranded anti-parallel sheet.

Despite these additions to the β-prism fold, the common core of the N-domain more closely resembles that of the well-studied and highly conserved monocot lectins (e.g. RMSD of 1.35 Å with snowdrop lectin compared to 1.82 Å for the C-domain). This structural divergence is in contrast with the degree of conservation of the carbohydrate-binding motif characteristic of the monocot lectins (QxDxNxVxY) in each of the three subdomains. In the N-domains of LlpA homologues, the surface-exposed motifs III and II are not well conserved and likely lost their function during evolution. In contrast they seem to be better conserved in the C-domains (Figure S4). Apparently, the two MMBL domains of LlpA experienced a differential evolutionary pressure resulting in different degrees of global and local (carbohydrate-binding motif) conservation, suggesting distinct functional roles for each domain.

The C-domain of LlpA_BW_ further extends into a β-hairpin that helps to define the relative orientations of its two MMBL domains. This β-hairpin is highly bent due to a β-bulge inserted into its second β-strand (Figure 1B). It is absent in all plant representatives including tandem MMBL proteins such as SCAfet (Figure 1C). In bacteria it represents the most divergent part of LlpA homologues, both in primary sequence and in length (Figure S5). Most of these C-terminal extensions terminate with a phenylalanine residue. This is reminiscent of the conserved terminal phenylalanine of outer membrane proteins from Gram-negative bacteria such as PhoE, required for their translocation to the cell envelope [39]. An equivalent extension appears to be absent in the *Xanthomonas* and *Arthrobacter* sequences (Figure S5).

### LlpA is capable of binding mannose-containing carbohydrates

**Figure 3:**
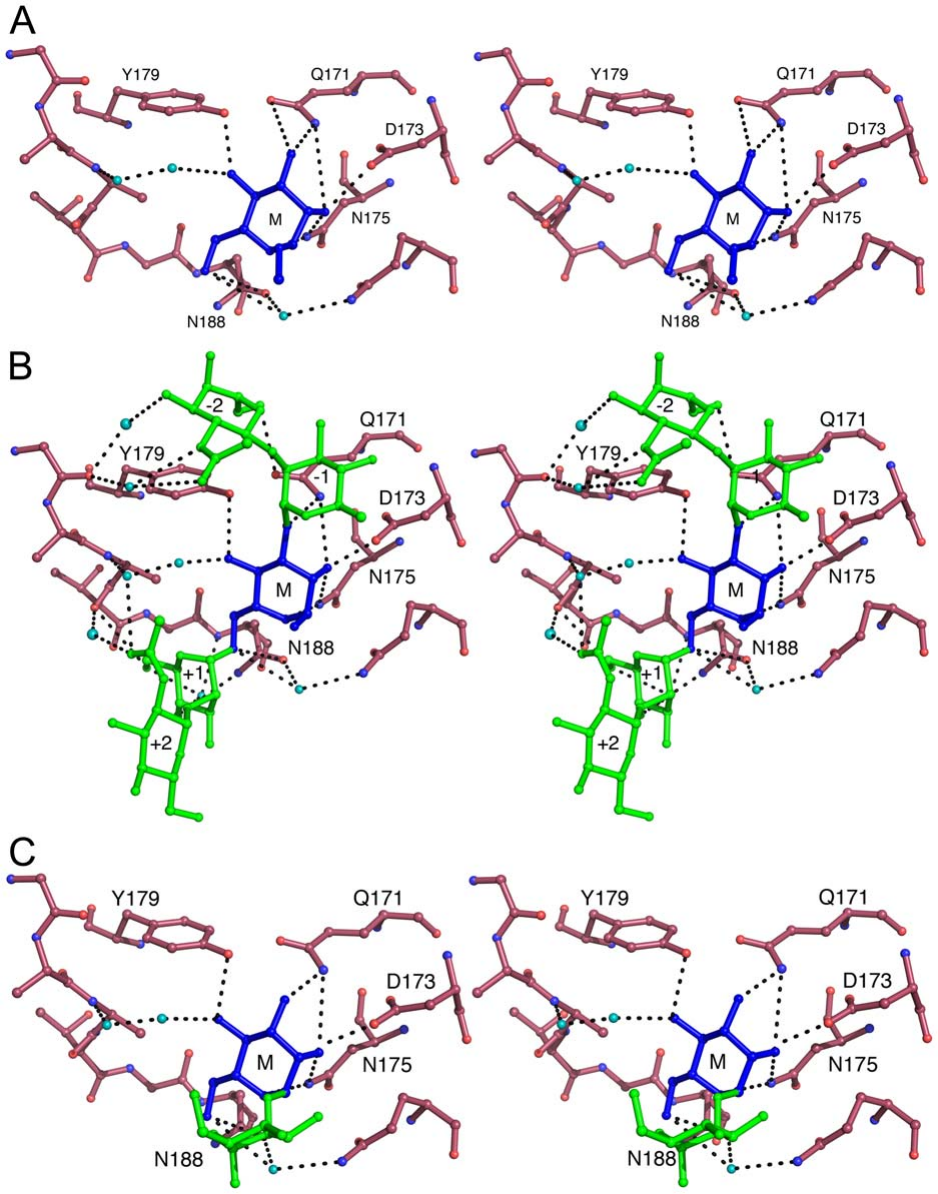
Carbohydrate binding in site III^C^ of LlpA_BW_. (A) Stereoview of methyl-α-D-mannopyranoside bound to subdomain III^C^. Methyl-α-D-mannopyranoside is shown in blue and indicated by M. Residues belonging to the QxDxNxVxY motif and hydrogen bonding to the sugar as well as Asn188 are labeled. Water molecules bridging protein and carbohydrate are shown in cyan (B) Similar view of the pentasaccharide GlcNAcβ(1–2)Manα(1–3)[GlcNAcβ(1–2)Manα(1–6)]Man. The mannose residue occupying the primary binding site is shown in blue and labeled M. The additional two mannoses (labeled +1 and −1) and two *N*-acetyl glucosamine residues (labeled +2 and −2) are shown in green. Other colors are as in panel A. (C) Binding of the disaccharide Manα(1–2)Man. The non-reducing mannose residue occupying the primary binding site is shown in blue and labeled M. The second, reducing mannose is shown in green. Other colors are as in panel A.

Subdomains II^C^ and III^C^of LlpA_BW_ contain the typical sugar-binding signature (QxDxNxVxY) of an active MMBL mannose-binding site (Figure S1 and S4). Soaking crystals of LlpA_BW_ with 200 mM methyl-α-D-mannopyranoside (Me-Man) led to clear electron density of a single Me-Man in site III^C^ of each of the two LlpA_BW_ monomers in the asymmetric unit (Figure S6A). This site comprises the side chains from Gln171, Asp173, Asn175 and Tyr179, which contribute to hydrogen bond interactions and the side chains of residues Val177, Asn188, Gln192 and Ala185, which contribute to van der Waals contacts with the carbohydrate ligand (Figure 3A, Figure S7A,C). This architecture is very similar to what is observed for mannose bound to other MMBL-type lectins such as snowdrop and garlic lectin (Figure S7B).

Soaks with oligomannoses revealed additional sugar-binding subsites. Binding site III^C^ accommodates the disaccharide Manα(1–2)Man and the pentasaccharide GlcNAcβ(1–2)Manα(1–3)[GlcNAcβ(1–2)Manα(1–6)]Man (Figure S6B,C). In the case of the pentasaccharide, the central reducing mannose is located in the shallow Me-Man binding site and the two GlcNAcβ(1–2)Man moieties stretch out over the surface making only a few additional hydrogen bonds or van der Waals contacts (Figure 3B). In the bound disaccharide, the non-reducing mannose is located in the Man-Me binding site while the reducing mannose faces the solvent and does not interact directly with the protein (Figure 3C).

Site II^C^ of both LlpA_BW_ molecules in the asymmetric unit is involved in crystal packing interactions and the presence of Me-Man is therefore sterically excluded. All residues that form specific hydrogen bonds with Me-Man are retained but substitutions occur for three side chains that provide van der Waals contacts (Figure S4 and S8A). In contrast, site I^C^ lost the conserved QxDxNxVxY motif (Figure S4) and is involved in inter-domain contacts and therefore inaccessible to ligands (Figure S8B).

The putative carbohydrate-binding sites in the N-domain of LlpA_BW_ are less conserved. Similar to the C-domain, site I^N^ is inaccessible and involved in inter-domain interactions (Figure S9A). In the II^N^ subdomain, the canonical mannose-binding motif QxDxNxVxY is essentially absent, with only the Gln residue of the motif being conserved as Gln82 (Figure S4). All other donors or acceptors required for hydrogen bonds with a mannose ligand are missing. In addition, the presence of Phe86 at the equivalent position of the expected Val sterically hinders the binding of mannose (Figure S9B). The potential carbohydrate-binding site on subdomain III^N^ is only partially conserved (Figure S9C) and contains two relevant substitutions from the canonical signature: (1) the Tyr residue of the QxDxNxVxY motif is replaced by the shorter Gln49, thereby removing the canonical hydrogen bond between Man O4 and Tyr OH, and (2) a threonine at position 54 which may compensate the hydrogen bond lost due to the Tyr-to-Gln substitution in the canonical motif. The lack of electron density at this site in our Me-Man soak nevertheless indicates that this site does not recognize this ligand or that its affinity is so low that recognition would only be achieved in the context of a larger and as yet unidentified mannose-containing ligand. Alternatively, this putative site may possess specificity for a different monosaccharide. In order to evaluate this hypothesis, we soaked LlpA_BW_ crystals with D-galactose, N-acetyl-D-glucosamine and L-fucose. No electron density was observed for any of these sugars, suggesting that the N-domain has a function distinct from carbohydrate recognition (data not shown).

### Carbohydrate-binding capacity is required for LlpA toxicity

The LlpA_BW_ motifs III^N^, III^C^ and II^C^ create potential carbohydrate binding sites that may be involved in bacteriotoxicity of the protein. We therefore examined the role of carbohydrate binding in the bactericidal function of LlpA_BW_. The presence of methyl-α-D-mannopyranoside up to 500 mM in the medium did not influence the activity of LlpA_BW_ on *P. syringae* GR12-2R3. Glycan array profiling did not highlight any specific oligosaccharide structure that could represent a natural ligand of LlpA_BW_ (Table S1). This could be due to the array design that is principally based on eukaryotic glycans and may therefore lack an appropriate carbohydrate for this prokaryotic toxin. Previously, it was observed that LlpA_BW_ from concentrated culture supernatant does not agglutinate rabbit red blood cells, nor binds to a mannose-agarose affinity matrix [21].

**Figure 4:**
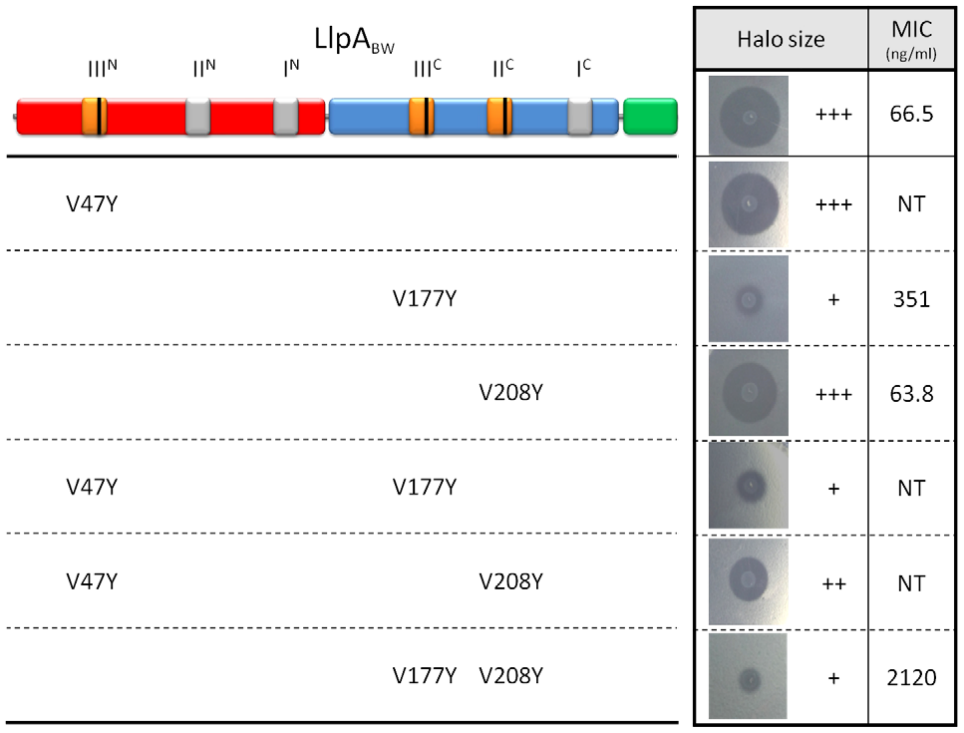
Inhibitory activity of wild-type LlpA_BW_ and selected mutants with modified (potential) mannose-binding sites. The domain structure (N-domain in red, C-domain in blue and C-terminal extension in green) and the position of the MMBL motifs (potentially active binding sites in orange, inactive ones in grey) are shown. The positions of conserved valine residues converted to tyrosine residues by site-directed mutagenesis are indicated with a black bar. Inhibitory activity of *E. coli* strains expressing mutant LlpA_BW_ forms was assayed against *P. syringae* GR12-2R3 and semi-quantified according to the size (inner zone radius) of the growth inhibition halo relative to LlpA_BW_ (+++, native LlpA_BW_; ++, halo size reduced; + halo size strongly reduced; −, no halo; NT, not tested). For wild-type LlpA_BW_ and three purified His-tagged mutant forms (LlpA_V177Y_, LlpA_V208Y_ and LlpA_V177Y-V208Y_) the MIC values were determined with indicator *P. syringae* GR12-2R3. Molar minimal inhibitory concentrations of recombinant proteins (with standard deviations): LlpA, 2.08 nM (±0.58 nM); LlpA_V177Y_, 10.9 nM (±0.66 nM); LlpA_V208Y_, 1.98 nM (±0.066 nM); 65.72 nM (±2.80 nM).

To assess whether the mannose-recognizing QxDxNxVxY motifs in LlpA_BW_ are nevertheless relevant for bactericidal activity, the conserved valine residue was mutated to tyrosine in subdomains III^N^, III^C^, and II^C^. These mutations sterically preclude mannose or any other ligand to enter the binding sites (Figure S7C). Semi-quantitative activity assays with permeabilized *E. coli* cells expressing the LlpA variants in motifs III^N^, III^C^ and II^C^ were used to assess the relationship between carbohydrate binding and bactericidal activity. Modification of the III^N^ site, for which no mannose binding was observed, does not affect the antibacterial activity against *P. syringae* GR12-2R3 (Figure 4). In contrast, the altered III^C^ pocket strongly diminishes activity, either alone or in pairwise combination with the other mutated sites (III^N^ or II^C^). A minor negative effect of the II^C^ mutation is only apparent in a double mutant, when combined with a modified III^N^ motif.

**Figure 5:**
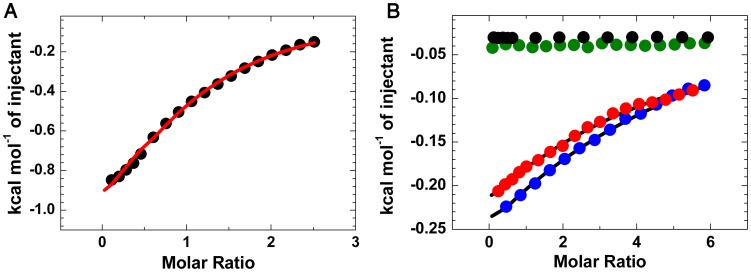
ITC analysis of carbohydrate binding to LlpA_BW_ and mutants. (A) Binding of LlpA_BW_ to the pentasaccharide GlcNAcβ(1–2)Manα(1–3)[GlcNAcβ(1–2)Manα(1–6)]Man. (B) Binding of LlpA_BW_ (blue circles, wild type) and the mutants LlpA_V177Y_ (green circles, site III^C^ knockout), LlpA_V208Y_ (red circles, site II^C^ knockout) and LlpA_V177Y-V208Y_ (black circles, site II^C^ and III^C^ knockout) to α-methyl mannoside. There is no heat exchanged in the titration of the double mutant or the site III^C^ knockout LlpA_V177Y_, whereas the site II^C^ knockout LlpA_V208Y_, binds the monosaccharide in a “wildtype”-like fashion, showing that only site III^C^ is involved in sugar binding.

Purified proteins were prepared to further quantify these effects. Far UV CD spectra of these mutant forms are identical to that of native protein LlpA_BW_, indicating that the mutations do not affect the overall structure of the protein. Isothermal titration calorimetry (ITC) showed that LlpA_BW_ has an affinity of 2.1 mM for the pentasaccharide GlcNAcβ(1–2)Manα(1–3)[GlcNAcβ(1–2)Manα(1–6)]Man, the highest among all the tested oligo-mannosides (See Figure 5 and Table 1 for a summary of the experimentally validated LlpA_BW_-carbohydrate interactions). This is in agreement with the crystal structures of the different complexes since this sugar is the one with the largest binding interface (Figure 3). Titrations of LlpA_BW_, of the mutants LlpA_V177Y_ (a site III^C^ knockout), LlpA_V208Y_ (a site II^C^ knockout) and of the double mutant LlpA_V177Y-V208Y_ with α-methyl mannoside clearly pinpoint site III^C^ as the only responsible for the sugar binding activity. Point mutations in both sites or III^C^ (V177Y) alone, completely abrogate sugar binding. However knocking out site II^C^ (V208Y) has little effect in binding and the affinities of LlpA_V208Y_ for α-methyl mannoside and Manα(1–3)Man are very close to the ones measured for the wild-type protein (See Table 1 and Figure 5B).

**Table 1:** Binding affinities and thermodynamic parameters obtained from ITC titrations.

Type of protein-carbohydrate interaction	K_d_ (mM)	ΔG°(kcal mol^−1^)	ΔH° (kcal mol^−1^)	−TΔS°(kcal mol^−1^)
LlpA_BW_ Me-α-D-Man	45.9	−1.8	−5.4	3.6
LlpA_BW_ Manα(1–2)Man	42.4	−1.9	−3.6	1.7
LlpA_BW_ Manα(1–3)Man	18.2	−2.4	−5.9	3.5
LlpA_BW_ Manα(1–6)Man	17.2	−2.4	−5.5	3.1
LlpA_BW_ Manα(1–3)[Manα(1–6)]Man	10.1	−2.6	−6.4	3.8
LlpA_BW_ GlcNAcβ(1–2)Manα(1–3)[GlcNAcβ(1–2)Manα(1–6)]Man	2.1	−3.7	−1.6	−2.1
LlpA_V208Y_ Me-α-D-Man	58.8	−1.7	−3.3	1.6
LlpA_V208Y_ Manα(1–3)Man	23.0	−2.2	−5.1	2.9

The reported values for K_d_, ΔG°, ΔH° and −TΔS° were determined from fitting a single site interaction model (n = 1) to the experimental ITC data. The interaction of the mutants LlpA_V177Y_ and LlpA_V177-V208Y_ with the different sugars is negligible and no heat effect was observed. Therefore they are not included in this table.

**Figure 6:**
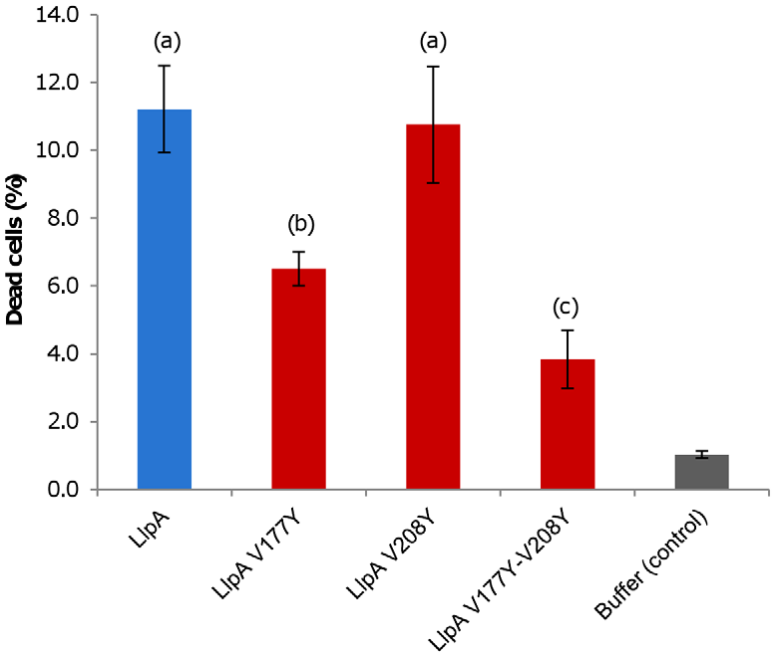
Killing activity of LlpA_BW_ and mutant proteins. Percentages of dead cells after live/dead staining as quantified by flow cytometry analysis (Figure S10). *P. syringae* GR12-2R3 was used as indicator strain and treated at a final concentration of 50 µg/ml for 1 h. Average values (with standard deviations; indicated by error bars): LlpA, 10.1 (±1.04); LlpA_V177Y_, 6.1 (±0.44); LlpA_V208Y_, 9.7 (±1.39); LlpA_V177Y-V208Y_, 3.7 (±0.90); buffer (control), 1.0 (±0.11).Values are significantly different for (a) and (b), (b) and (c) (p<0.01).

While the V208Y mutation in the II^C^ site has no observable effect on the MIC value for *P. syringae* GR12-2R3, the altered III^C^ motif engenders a 5.2-fold increase in MIC (Figure 4). The mutant protein LlpA_V177Y-V208Y_ suffers a further reduction in activity, yielding a 31.6-fold increased MIC compared to native LlpA_BW_. The biological activities of LlpA and its mutants were further assessed by live/dead staining and subsequent flow cytometry analysis (Figure 6, Figure S10). Proportions of dead cells after 1 hour of exposure to LlpA or LlpA_V208Y_ were comparable (10.1% and 9.7%, respectively). For LlpA_V177Y_ this value was reduced to 6.1%, significantly lower than for LlpA. Killing activity was even further reduced for LlpA_V177Y-V208Y_ (3.7%). These results are consistent with the MIC determination and ITC data, indicating that an active site III^C^ is required to generate a fully active LlpA bacteriocin. The difference in bacteriotoxicity between LlpA_V177Y_ and LlpA_V177Y-V208Y_ suggests that site II^C^ has a supporting role in the LlpA_BW_ bacteriotoxicity.

### All domains are necessary for LlpA_BW_ functionality

The site-directed mutagenesis approach revealed an important role for the C-domain's carbohydrate-binding capacity in LlpA_BW_ toxicity. Considering the increased binding motif degeneration in the N-domain and the fact that a *Ruminococcus* bacteriocin composed of only a single MMBL domain fused to an unknown domain has been identified [35], the N-domain may fulfill a distinct function, different from that of the C-domain. In order to scrutinize the contribution of individual domains to overall activity, six domain deletion constructs of *llpA_BW_* were engineered to potentially encode proteins lacking the first or second MMBL domain, a gene product devoid of the C-terminal hairpin, or a protein retaining only an individual domain (N-domain, C-domain, or hairpin) (Figure S11). To take the domain swapping into account, the constructs containing only a single MMBL domain were designed with a fusion of the swapped C-terminal β-strands to the corresponding domain via a short linker.

**Figure 7:**
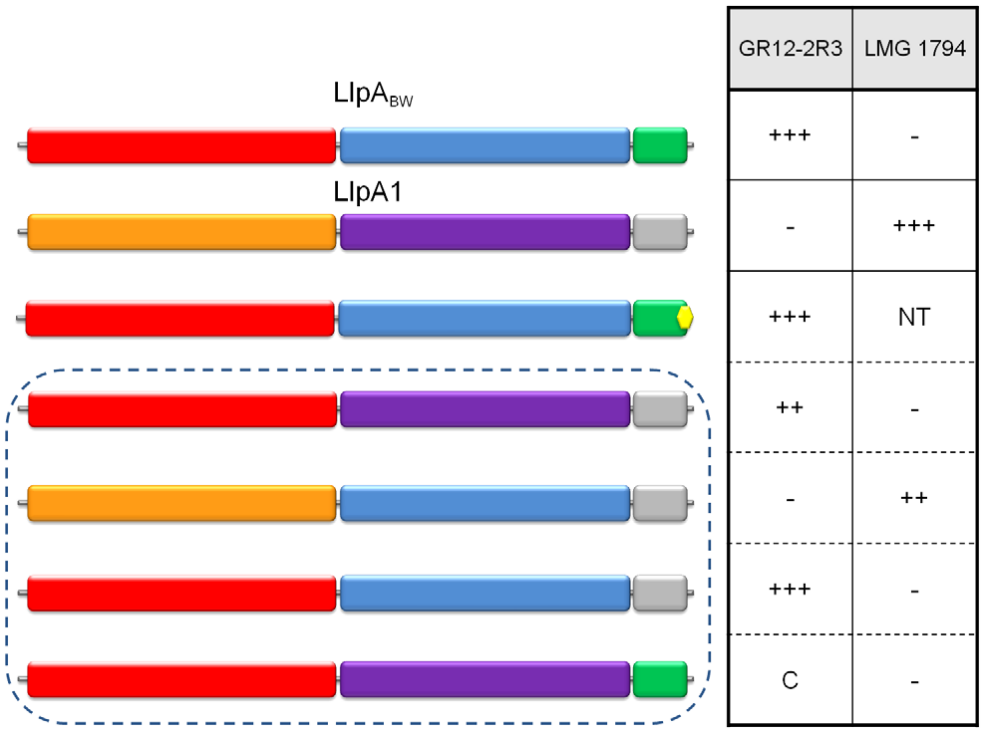
Differential inhibitory activity of wild-type LlpA_BW_ and LlpA/LlpA1 domain chimers. The domain structures of LlpA_BW_ (as in Figure 4) and of LlpA1 (inferred by pairwise alignment; N-domain in orange, C-domain in purple and C-terminal extension in grey) are depicted, along with those of chimeric forms (in dashed box). The LlpA variant lacking the terminal phenylalanine residue is marked with a yellow hexagon. Inhibitory activity of the respective *E. coli* recombinants was tested with diagnostic indicators for LlpA_BW_ (*P. syringae* GR12-2R3) and LlpA1 (*P. fluorescens* LMG 1794). Halo sizes are semi-quantified according to size of the growth inhibition halo (+++, native halo size of LlpA_BW_ and LlpA1; ++, halo size reduced; C, local clearing confined to producer colony spot; −, no halo or clearing; NT, not tested). Additional chimeric and domain deletion constructs not conferring bacteriocin activity against one of the indicator strains are specified in Figure S11.

None of these deletion constructs resulted in the production of an active protein, indicating that none of the domains are dispensable. Removal of the terminal phenylalanine residue still allows expression of a functional bacteriocin in *E. coli* (Figure 7), but a further C-terminal truncation (deletion of Trp-His-Phe tail) resulted in a negative bacteriocin assay (data not shown). From these data we conclude that both MMBL domains as well as the C-terminal hairpin extension are required for activity of LlpA. Whether the role of the C-terminal hairpin is any other than simply stabilization of the C-domain cannot be concluded.

### Target specificity of LlpA is hosted by the N-domain

In order to investigate the role of the different domains in target specificity, we created hybrid LlpA proteins using the domains of LlpA_BW_ from *P. putida* BW11M1 and LlpA1 from *P. fluorescens* Pf-5. These two LlpA proteins share 45% sequence identity and differ in their target spectra. Strains *P. syringae* GR12-2R3 and *P. fluorescens* LMG1794 were identified as specific indicators for LlpA_BW_ [21] and LlpA1 [22], respectively. Six constructs carrying *llpA_BW_*/*llpA1*chimeric genes were made with domain exchanges involving the N-domain, C-domain, and hairpin region (Figure 7 and Figure S11). For four of these constructs activity against one of both indicators was detected. Only constructs retaining the original N-domain give rise to inhibition of the cognate indicator strain. The C-domain or the hairpin of LlpA_BW_ could be replaced with the corresponding LlpA1 domains without changing target specificity. Conversely, the original specificity of LlpA1 is retained upon replacement of its C-domain by the LlpA_BW_ equivalent.

## Discussion

Structure elucidation of LlpA_BW_ from *P. putida* BW11M1 unequivocally assigns this bacteriocin to the MMBL lectin family, in which it constitutes the first prokaryotic member, representative for a group of bacterial proteins composed of two MMBL domains [21]–[23]. Systematic inactivation of the three potential carbohydrate-binding sites present in the N-domain (III^N^) and in the C-domain (III^C^ and II^C^) of LlpA_BW_, revealed that a non-occluded III^C^ pocket is required to obtain a fully active LlpA_BW_ molecule. A negative co-operative effect on activity resulted when the II^C^ site was additionally modified. Although mannose-containing carbohydrates can bind to the III^C^ pocket of LlpA_BW_, it remains unclear if these are part of or mimic the natural ligand required for biological activity since bacteriocin activity is not impaired in the presence of excess mannose.

A mutated III^N^ site did not provoke a negative effect on antibacterial activity. However, the N-domain appears to play a major role in target selection. This was demonstrated by assessing the differential activity of domain chimers against two target strains, diagnostic for the LlpA_BW_- and LlpA1-specific killing.

The β-hairpin does not appear to be a specificity determinant, although it constitutes the most variable region among LlpA-like bacteriocins. Possibly, it is required for thermodynamic stability since it needs to be intact in LlpA_BW_. An equivalent C-terminal stretch is absent from the *Xanthomonas citri* LlpA-like bacteriocin [23]. From our results relying on heterologous expression in *E. coli* and a bacteriocin assay with permeabilized cells, it cannot be excluded that this structural element may play a role in the way an LlpA protein is exported by its native producer cells.

In general, a defensive role has been proposed for the (oligo)mannose-binding MMBL lectins based on insecticidal, nematicidal, antifungal, or even antiviral activities demonstrated for several of these proteins that are abundantly found in monocot plants [40]–[46]. Some of these plant lectins trigger apoptosis in cancer cells [47]. Also their identification in fish mucus and epithelial cells is in line with a general protective (antimicrobial) function for MMBL domains [32]. LlpA_BW_ as a bactericidal protein fits within this picture of MMBL domains being involved in general defense mechanisms. Since no antibacterial activity has been assigned to the eukaryotic MMBL proteins, it is challenging to identify structural features that confer the intragenus-specific bacteriocin activity of LlpA, as shown for proteins from *P. putida* [21], *P. fluorescens* [22], *P. syringae* [23], and *Xanthomonas citri* [23]. Their target spectra are narrower than reported for the mammalian antibacterial C-type lectins of the RegIII family, such as mouse RegIIIγ and its human homolog HIP/PAP that bind to the surface-exposed peptidoglycan layer of Gram-positive bacteria [48], and RegIIIβ that also binds to the lipid A moiety of lipopolysaccharides on the cell envelope of Gram-negative bacteria [49].

The absence of any known secretory signal sequence in LlpA_BW_ and its homologues in other *Pseudomonas* species is intriguing in view of their extracellular location [21]. The translocation of the outer membrane-associated mannose/fucose-specific lectin LecB of *P. aeruginosa*, that also lacks such signal sequence [50], is dependent on its glycosylation [51]. Contrary to LlpA that is exported to the culture supernatant to exert its antagonistic activity, LecB remains associated with the cell envelope through interaction with the major outer membrane protein OprF [52], in line with its role in biofilm formation.

## Materials and Methods

### Strains and culture conditions

Bacterial strains and plasmids used in this study are listed in Table S2. *Escherichia coli* was routinely grown in shaken Luria-Bertani (LB, MP Biomedicals) broth at 37°C. *Pseudomonas* strains were grown in Tryptic Soy Broth (BD Biosciences) at 30°C with shaking. Media were solidified with 1.5% agar (Invitrogen) and supplemented with filter-sterilized antibiotics as required at following concentrations: ampicillin (Sigma-Aldrich), 100 µg/ml or kanamycin (Sigma-Aldrich), 50 µg/ml. Isopropyl β-D-thiogalactoside (IPTG 40 µg/ml, ForMedium) and 5-bromo-4-chloro-3-indolyl-β-D-galactopyranoside (X-Gal 40 µg/ml, ForMedium) were added for blue/white screening of pUC18-derived plasmids in *E. coli*.

Plasmids used for antibacterial testing and sequencing were propagated in *E. coli* TOP10F′ (Invitrogen). *E. coli* BL21(DE3) (Novagen) was used as a host for plasmids driving recombinant protein expression. Genomic DNA from *P. putida* BW11M1 was isolated using the Puregene Yeast/Bact. Kit B (Qiagen). Plasmid DNA was extracted using the QIAprep Spin Miniprep Kit (Qiagen). Stocks were stored at −80°C in the appropriate medium in 25% (v/v) glycerol.

### Recombinant DNA methods

Standard methods were used for preparation of competent *E. coli* cells, heat shock transformation of *E. coli* and DNA electrophoresis [53]. Restriction enzymes were used according to the supplier's specifications (Roche Diagnostics and BIOKÉ). DNA ligation was performed using T4 DNA ligase (Invitrogen). Plasmid sequencing was performed by GATC Biotech (Constance, Germany). Constructs that were generated are listed in Table S2 and primers are listed in Table S3.

A 921-bp fragment containing *llpA_BW_* was amplified by PCR with Platinum *Pfx* DNA polymerase (Invitrogen), using a C1000 Thermal Cycler (Bio-Rad). *P. putida* BW11M1 genomic DNA was taken as a template, and combined with primers PGPRB-3155 and PGPRB-3156. The amplicon was purified using the QIAquick PCR Purification Kit (Qiagen), digested with KpnI and BamHI, ligated in pUC18, and transformed to *E. coli* TOP10F′. Transformants were verified for the presence of the insert by PCR using Taq Polymerase (BIOKÉ) with primers PGPRB-2545 and PGPRB-2546. The cloned construct (pCMPG6129) was purified and its insert confirmed by sequencing.

DpnI-mediated site-directed mutagenesis was performed to construct valine-to-tyrosine mutant forms of *llpA_BW_* in pUC18 (pCMPG6129) and N-terminal His-tagged *llpA_BW_* in pET28a (pCMPG6056 [54]). PCR conditions were: 2 min initial denaturation, followed by 16 cycles of denaturation (1 min), annealing (1 min, primer-dependent temperature) and elongation at 68°C (1 min./kb). Final elongation was for 8 min at 68°C. After PCR, samples were immediately treated with DpnI at 37°C for 2 h and transformed into *E. coli* TOP10F′ and selected on the appropriate medium. Plasmid inserts of selected transformants were verified by sequence analysis. Double mutants were constructed using plasmids with a single point mutation as a template.

Domain deletants of *llpA_BW_* were constructed using pCMPG6129 as a template (*llpA_BW_* from *P. putida* BW11M1). Chimeric constructs were obtained using pCMPG6129 and pCMPG6053 (*llpA1* from *P. fluorescens* Pf-5 [22]) as templates. Artificial ligation of gene fragments, generated with the PCR primers specified in Table S3, was performed by using splicing by overlap extension (SOE). The resulting recombinant amino acid sequences are listed in Table S4.

### Recombinant protein expression and purification

Protein isolation and purification of N-terminal His_6_-tagged LlpA_BW_, LlpA_V177Y_, LlpA_V208Y_, and LlpA_V177Y-V208Y_ from *E. coli* BL21(DE3), carrying expression constructs pCMPG6056, pCMPG6149, pCMPG6150 and pCMPG6151 respectively, were performed as described by Parret and collaborators [54]. The presence of His-tagged protein was observed via immunodetection by Western blot, using monoclonal anti-His_6_ (IgG1 from mouse; Roche Diagnostics) as primary antibody. Fractions free of other proteins, as verified by SDS-PAGE and subsequent Coomassie Blue staining, were dialyzed against bis-TRIS propane buffer (20 mM, 200 mM NaCl, pH 7.0). Concentrations of purified proteins were determined by absorbance measurement at 280 nm using molar extinction coefficients of 62910 M^−1^ cm^−1^ for LlpA_BW_, 64400 M^−1^ cm^−1^ for LlpA_V177Y_ and LlpA_V208Y_, and 65890 M^−1^ cm^−1^ for LlpA_V177Y-V208Y_. Extinction coefficients were calculated according to Pace and collaborators [55].

### Antibacterial assays

Bacteriocin production by *E. coli* cells carrying pUC18-derived constructs was assayed as follows: 2-µl drops of an overnight stationary-phase culture were spotted onto LB agar plates and incubated for 8 h at 37°C. Next, plates were exposed to chloroform vapor (30 min), aerated and subsequently overlaid with 5 ml of soft agar (0.5%), seeded with 200 µl of a cell culture of an indicator strain (∼10^8^ CFU/ml), followed by overnight incubation at 30°C. Next day, plates were scored for the presence of halos in the confluently grown overlay.

Antibacterial activity assays with purified recombinant His_6_-tagged proteins were performed as described by Ghequire and collaborators [23]. To assess the influence of added sugar, the same assay was carried out on agar medium supplemented with D-mannose (Sigma-Aldrich) or methyl-α-D-mannopyranoside (Sigma-Aldrich) to a final concentration of 0.01 M to 0.5 M.

A Bioscreen C apparatus (Oy Growth Curves Ab Ltd, Finland) was used to determine the minimum inhibitory concentration (MIC). An overnight culture (16 h) of the indicator strain was diluted to 10^4^–10^5^ CFU/ml and incubated at 30°C, with a two-fold dilution series of recombinant His_6_-tagged LlpA_BW_ or mutant LlpA_BW_. Bis-TRIS propane buffer was used as control. The MIC value was determined as the minimum concentration of protein at which no growth of the indicator strain (OD_600_<0.2) occurred after 24 h. At least three independent repeats, each with three replicates, were carried out.

### Glycan array

His_6_-tagged LlpA_BW_ was lyophilized and verified for antibacterial activity. After re-dissolving in MilliQ water, recombinant LlpA_BW_ was diluted to 200 µg/ml with binding buffer (20 mM TRIS-HCl pH 7.4, 150 mM NaCl, 2 mM CaCl_2_, 2 mM MgCl_2_, 0.05% Tween 20, 1% BSA), and used to probe the printed glycan arrays [56] following the standard procedures of Core H of the Consortium for Functional Glycomics (http://www.functionalglycomics.org/). Monoclonal anti-His_6_ antibodies (Roche Diagnostics) were used as primary antibodies, and fluorescently labeled anti-mouse IgG as secondary antibodies.

### Circular dichroism

CD spectra were acquired on a Jasco J-715 spectropolarimeter. Curves were averaged over 8 scans, taken at 25°C using a 1 mm cuvette. Samples were dialyzed against bis-TRIS propane buffer (20 mM, NaCl 200 mM, pH 7.0), filtered and degassed prior to data acquisition. All proteins were assayed at 0.4 mg/ml.

### Isothermal titration calorimetry

ITC titrations were carried out on an ITC200 apparatus (MicroCal). Prior to the measurement, LlpA_BW_, LlpA_V177Y_, LlpA_V208Y_ and LlpA_V177Y-V208Y_ was dialyzed to bis-TRIS propane buffer. Sugars were directly dissolved into the same buffer. The samples were filtered and degassed for 10 min at 25°C before being examined in the calorimeter. The titrations were carried out at 25°C, injecting the sugars (methyl-α-D-mannoside, Manα(1–2)Man, Manα(1–3)Man, Manα(1–6)Man, Manα(1–3)[Manα(1–6)]Man and GlcNAcβ(1–2)Manα(1–3)[GlcNAcβ(1–2)Manα(1–6)]Man) into a protein solution (protein concentrations ranged from 2 mM to 4 mM depending on protein availability). All data were analyzed using the MicroCal Origin ITC 7.0 software. Binding affinities and thermodynamic parameters from all ITC titrations are reported in Table 1.

### X-ray data collection and structure determination

Expression, purification and crystallization of recombinant His-tagged LlpA_BW_ have been described [54]. X-ray data for native and derivative crystals were collected on EMBL beamline BW7A of the DESY synchrotron (Hamburg, Germany). For each potential derivative, the wavelength was chosen to be at the high-energy side of the absorption edge in order to ensure a usable anomalous signal. All data were scaled and merged with the HKL package of programs. Data collection statistics are given in Table S5.

The crystal structure of free LlpA_BW_ was solved combining single isomorphous replacement with anomalous scattering (SIRAS strategy) from a *p*-chloromercurybenzoate derivative. The heavy-atom substructure was determined with SHELXD [57] using a resolution cutoff of 4.0 Å. Heavy-atom refinement and phasing were performed with SHARP [58]. Phase improvement by solvent flattening was performed with SOLOMON [59]. Non-crystallographic symmetry averaging with density modification [60] further improved the electron density. A partial model (94% of the residues comprising the asymmetric unit) was automatically built with ARP/wARP [61] and the remainder was built manually over several cycles of model building with Coot [62], alternated with refinement using *phenix.refine* [63], [64]. Phasing and refinement statistics are shown in Table S5.

### Carbohydrate soaks

Crystals of LlpA_BW_ were transferred to artificial mother liquor (0.1 M imidazole pH 6.5, 1.3 M sodium acetate) enriched with either 200 mM methyl-α-D-mannopyranoside (Me-Man), Manα(1–2)Man, GlcNAcβ(1–2)Manα(1–3)[GlcNAcβ(1–2)Manα(1–6)]Man (M592), D-galactose, L-fucose or *N*-acetyl-D-glucosamine and allowed to equilibrate overnight (all carbohydrates obtained from Dextra Laboratories, Reading, U.K.). Data were collected at room temperature on EMBL beamline X13, except for the *N*-acetyl-D-glucosamine soak collected at the PROXIMA-1 beamline of the SOLEIL synchrotron (Gif-sur-Yvette, France) and the D-galactose soak collected at ESRF beam line ID14-1 (Grenoble, France). All data were scaled and merged using the HKL package. Refinement was started from the coordinates of the ligand-free structure using *phenix.refine*. Manual rebuilding, including the introduction of the carbohydrate ligand if present, was done with Coot [62]. Crystal structures of LlpA_BW_ from *P. putida* BW11M1 (PDB entry 3M7H) and LlpA_BW_ in complex with methyl-α-D-mannoside (PDB entry 3M7J), with Manα(1–2)Man (PDB entry 4GC1), and with GlcNAcβ(1–2)Manα(1–3)[GlcNAcβ(1–2)Manα(1–6)]Man (PDB entry 4GC2) have been deposited at the PDB.

### Flow cytometry

Overnight cultures of *P. syringae* GR12-2R3 (16 h) were diluted to OD_600_ 0.5 and washed twice with phosphate-buffered saline (PBS). Cells were treated with LlpA, mutant proteins or buffer (bis-TRIS propane buffer, negative control), at a final concentration of 50 µg/ml for 1 h, at 20°C. Next, PBS-washed bacteria were stained using the Live/Dead BacLight bacterial viability kit (Invitrogen), incubated for 15 minutes, and analyzed on a BD Influx (BD Biosciences). Excitation of the dyes was done at 488 nm, and fluorescence measured at 530 nm for SYTO 9 and at 610 nm for propidium iodide. Results were processed with FlowJo 10.0.4 software (Figure S10). Measurements were done independently and based on six biological repeats. Results are expressed as percentages of dead cells [dead/(live+dead) * 100)].

## Supporting Information

Figure S1 **Amino acid sequence of LlpA_BW_ colored according to its domain structure.** The N-domain is shown in red, the C-domain in blue and the C-terminal extension in green. Residues belonging to sequences equivalent to the mannose binding site signature motif QxDxNxVxY are in bold and underlined.
(JPG)

Figure S2 **Quaternary structures and domain organization of various MMBL family members.** Individual domains or protomers are shown in different colours. The domain or protomer colored green (which in the tandem MMBLs of LlpA, ASA I and SCAfet corresponds to the N-terminal domain) is always shown in the same orientation. Bound carbohydrates are shown in black stick representation. For LlpA a single pentasaccharide is bound to site III^C^. In the case of *Galanthus nivalis* (snowdrop) lectin (PDB entry 1JPC), twelve trimannosides are bound to all QxDxNxVxY motifs (three on each monomer of the homotetrameric protein). The snowdrop lectin tetramer consists of the association of two domain-swapped dimers (green-blue and pink-yellow). In the case of *Allium sativum* (garlic lectin ASA I - PDB entry 1KJ1), again each QxDxNxVxY motif has a dimannose bound while an additional sugar (shown in red) is bound to a non-canonical site. The protein is synthesized as a single chain precursor and post-translationally cleaved into two MMBL domains that adopt the same domain-swapped dimer as found in snowdrop lectin. Gastrodianin is a monomeric MMBL family member from the orchid *Gastrodia elata* (PDB entry 1XD5). The location(s) of its carbohydrate-binding site(s) is (are) not known. The fetuin-binding tandem-MMBL SCAfet from *Scilla campanulata* (PDB entry 1DLP) consists of two covalently attached MMBL domains, whereas in LlpA the swap of the C-terminal β-strands is retained. The relative orientation in the two domains is as in ASA I. This lectin binds fetuin rather than oligomannosides, but the locations of the binding sites are not known.
(JPG)

Figure S3 **Stereo view of the superpositions (Cα representations) of the N-domain of LlpA_BW_ (red), C-domain of LlpA_BW_ (blue) and** ***Galanthus nivalis*** **lectin (PDB entry 1MSA, black).** The superposition is shown in two orientations rotated by 90°.
(JPG)

Figure S4 **Sequence alignment of potential mannose-binding motifs in prokaryotic tandem MMBL proteins.** The sequences corresponding to the consensus motif QxDxNxVxY, extracted from the N-domain and the C-domain of *P. putida* LlpA_BW_ and its homologues, are aligned per domain. Sequence conservation is visualized by differential shading. The sequence logo representation visualizes the degree of consensus for each residue. LlpA proteins with proven bacteriotoxic activity are labeled with an asterisk. Accession numbers: *Arthrobacter* sp. FB24 (YP_829274), *Burkholderia ambifaria* MEX-5 (ZP_02905572), *Burkholderia cenocepacia* AU 1054 ([1], ABF75998; [2], ABF75999), *Pseudomonas chlororaphis* subsp. *aureofaciens* 30–84 (EJL08681), *Pseudomonas putida* BW11M1 (AAM95702), *Pseudomonas fluorescens* Pf-5 (LlpA1 [1], YP_258360; LlpA2 [2], YP_259234), *Pseudomonas* sp. GM80 ([1], ZP_10606046; [2], ZP_10606131), *Pseudomonas syringae* pv. *aptata* DSM 50252 (EGH77666), *Pseudomonas syringae* pv. *syringae* 642 (ZP_07263221), *Xanthomonas axonopodis* pv. *citri* str. 306 (AAM35756).
(TIF)

Figure S5 **Sequence alignment of the carboxy-terminal sequences of LlpA-like proteins.** The *P. putida* LlpA_BW_ sequence adopting a β-hairpin fold is delineated in Figure S1. The preceding conserved tryptophan residue is located C-terminally to I^C^ (Figure S1). The sequence logo representation visualizes the degree of consensus for each residue. Accession numbers are listed in Figure S4.
(TIF)

Figure S6 **Electron density for (A) Methyl-α-D-Man, (B) Manα(1–2)Man and (C) GlcNAcβ(1–2)Manα(1–3)[GlcNAcβ(1–2)Manα(1–6)]Man.** Difference electron-density maps are calculated by removing the sugar residues from the final coordinates and applying one round of slow-cool simulated annealing refinement to remove potential bias. The atomic model is superimposed in each case.
(JPG)

Figure S7 **Mannose binding to LlpA_BW_ and garlic lectin.** (A) Cartoon representation of subdomain III^C^ of LlpA_BW_ (green) with residues implicated in carbohydrate binding showing in ball-and-stick representation and labeled (carbon green, oxygen red, nitrogen blue). The Me-Man residue is shown in red. Selected hydrogen bonds are shown as black dotted lines. (B) Stereoview of the superposition of subdomain III^C^ of LlpA_BW_ (green) on the equivalent subdomain of garlic lectin (blue). The Me-Man residue bound to LlpA_BW_ is shown in red, the mannose bound to garlic lectin in blue. (C) Stereoview of the superposition of subdomain III^C^ of LlpA_BW_ (green) identical as in panel A, but emphasizing the location of Val177 (shown as black sticks). The modeled Val177Tyr mutation is shown as orange sticks. Tyr177 makes a steric clash with the bound mannose (red) and is therefore expected to prevent binding, in agreement with our ITC experiments.
(JPG)

Figure S8 **Sites II and I of the LlpA_BW_ C-domain.** (A) Stereoview of site II^C^ of the C-domain (colored according to atom type) superimposed on site III^C^ of the C-domain (dark gray). The Me-Man bound in site III^C^ is shown in red. This site is very similar to site III^C^ but in the crystal it is inaccessible due to crystal lattice interactions. Residue labels correspond to residues of site II^C^. (B) Similar view showing site I^C^ of the C-domain (colored according to atom type) superimposed on site III^C^ of the C-domain (dark gray). The Me-Man bound in site III^C^ is shown in red. The stretch of Ile137-Leu139 that provides a steric conflict preventing Me-Man binding in site I^C^, is highlighted with carbon atoms drawn in green. Residue labels correspond to residues of site I^C^.
(JPG)

Figure S9 **Sites of the LlpA_BW_ N-terminal domain.** (A) Stereoview of site I^N^ of the N-domain (colored according to atom type) superimposed on site III^C^ of the C-domain (dark gray). The Me-Man bound in site III^C^ is shown in red. The stretch of Ile271-Trp274 that provides a steric conflict preventing Me-Man binding in site I^N^ is highlighted with carbon atoms drawn in green. Residue numbering corresponds to residues of site I^N^. (B) Similar superposition for site II^N^ of the N-domain. Phe86 that prevents Me-Man binding to this site through a steric conflict is highlighted in green. Other residues belonging to site II^N^ are labeled in teal. Three residues of site III^C^ for which site II^N^ has no structural equivalent are labeled in black. (C) Similar superposition for site III^N^ of the N-domain. Residues belonging to site III^N^ are labeled in teal. One residue of site III^C^ for which site III^N^ has no structural equivalent, is labeled in black. For this site there are no obvious steric conflicts that would prevent positioning of a Me-Man residue although none is observed experimentally.
(JPG)

Figure S10 **Quantification of live and dead cells by flow cytometry.** *P. syringae* GR12-2R3 cells were treated with LlpA (A), LlpA_V177Y_ (B), LlpA_V208Y_ (C), LlpA_V177Y-V208Y_ (D), or buffer (E) at a final concentration of 50 µg/ml for 1 h at 20°C. After live/dead staining, cells were analysed by flow cytometry. Data processing allowed to distinguish populations of dead (left) and live (right) cells. Spot densities ranging from high to low are differentiated by a color gradient from red, yellow, green, teal to blue. Representative samples for LlpA, mutant proteins and buffer control are shown in panels A–E.
(TIF)

Figure S11 **Overview of inactive LlpA_BW_ deletants and inactive LlpA_BW_/LlpA1 chimers.** The equivalent domains of LlpA1 are delineated based on pairwise sequence alignment with LlpA_BW_: N-domain (orange), C-domain (purple), C-terminal extension (grey). No bacteriocin activity was conferred by these constructs upon recombinant *E. coli* cells tested against *P. syringae* GR12-2R3 (indicator strain for native LlpA_BW_) and *P. fluorescens* LMG 1794 (indicator strain for native LlpA1). The small black rectangle represents an artificial linker sequence (DASRS).
(TIF)

Table S1 **Glycan array profile of LlpA_BW_ as measured by fluorescence intensity.** Results including a comprehensive list of oligosaccharides (array version PA_v5) are available from the Consortium of Functional Glycomics (CFG, www.functionalglycomics.org).
(XLS)

Table S2 **Bacterial strains and plasmids used in this study.**
(DOC)

Table S3 **PCR primers used in this study.**
(DOCX)

Table S4 **Protein sequences of LlpA_BW_ deletants and LlpA_BW_/LlpA1 chimers.**
(DOCX)

Table S5 **Structure determination and refinement.**
(DOCX)
