# Supplementary figures and images for: Structural Determinants for Activity and Specificity of the Bacterial Toxin LlpA
Source: PLoS Pathog. 2013 Feb 28;9(2):e1003199. doi: 10.1371/journal.ppat.1003199 (PMC3585409; doi:10.1371/journal.ppat.1003199)

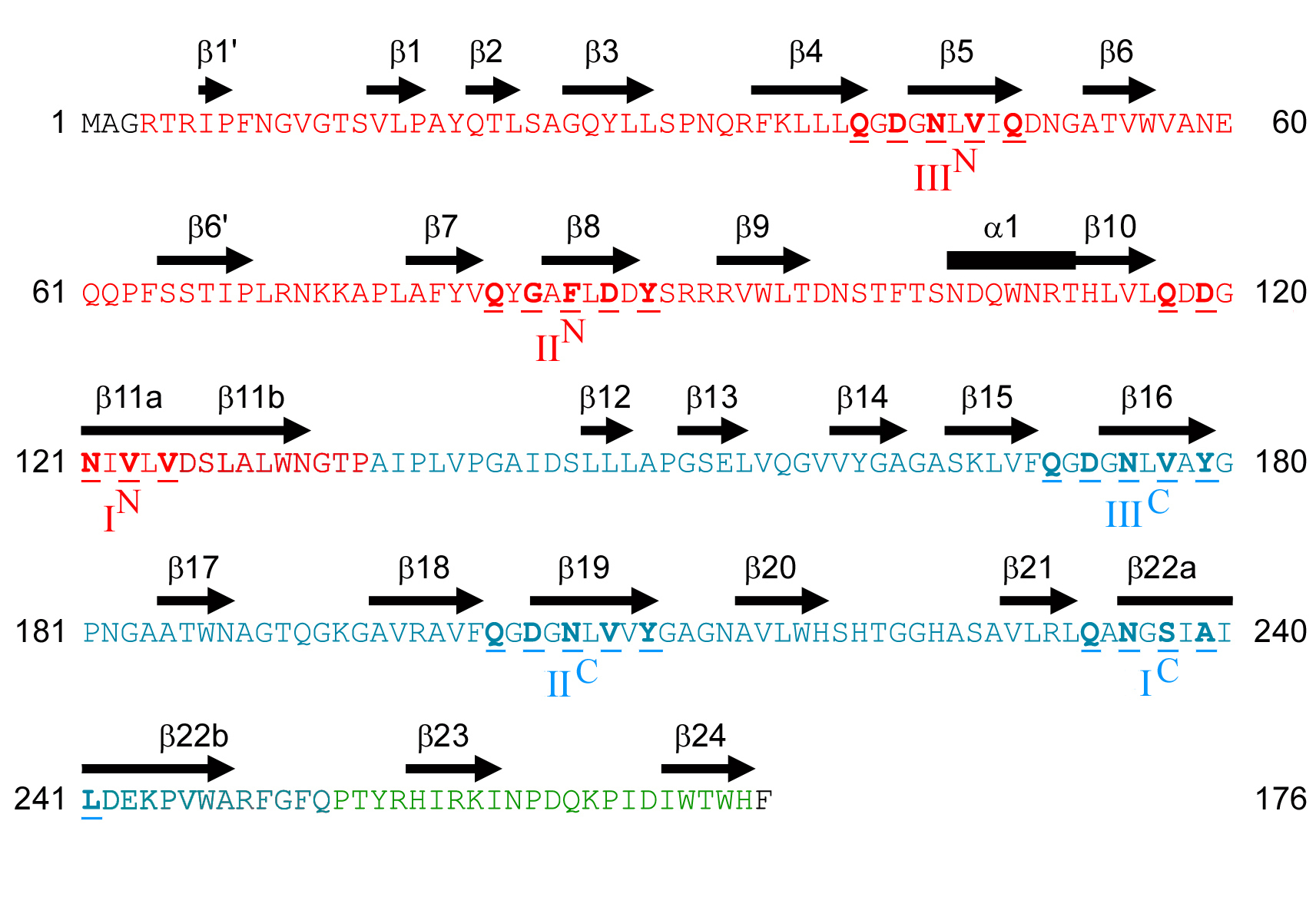

Supplement: Figure S1 — Amino acid sequence of LlpABW colored according to its domain structure. The N-domain is shown in red, the C-domain in blue and the C-terminal extension in green. Residues belonging to sequences equivalent to the mannose binding site signature motif QxDxNxVxY are in bold and underlined. (JPG) [file ppat.1003199.s001.jpg]

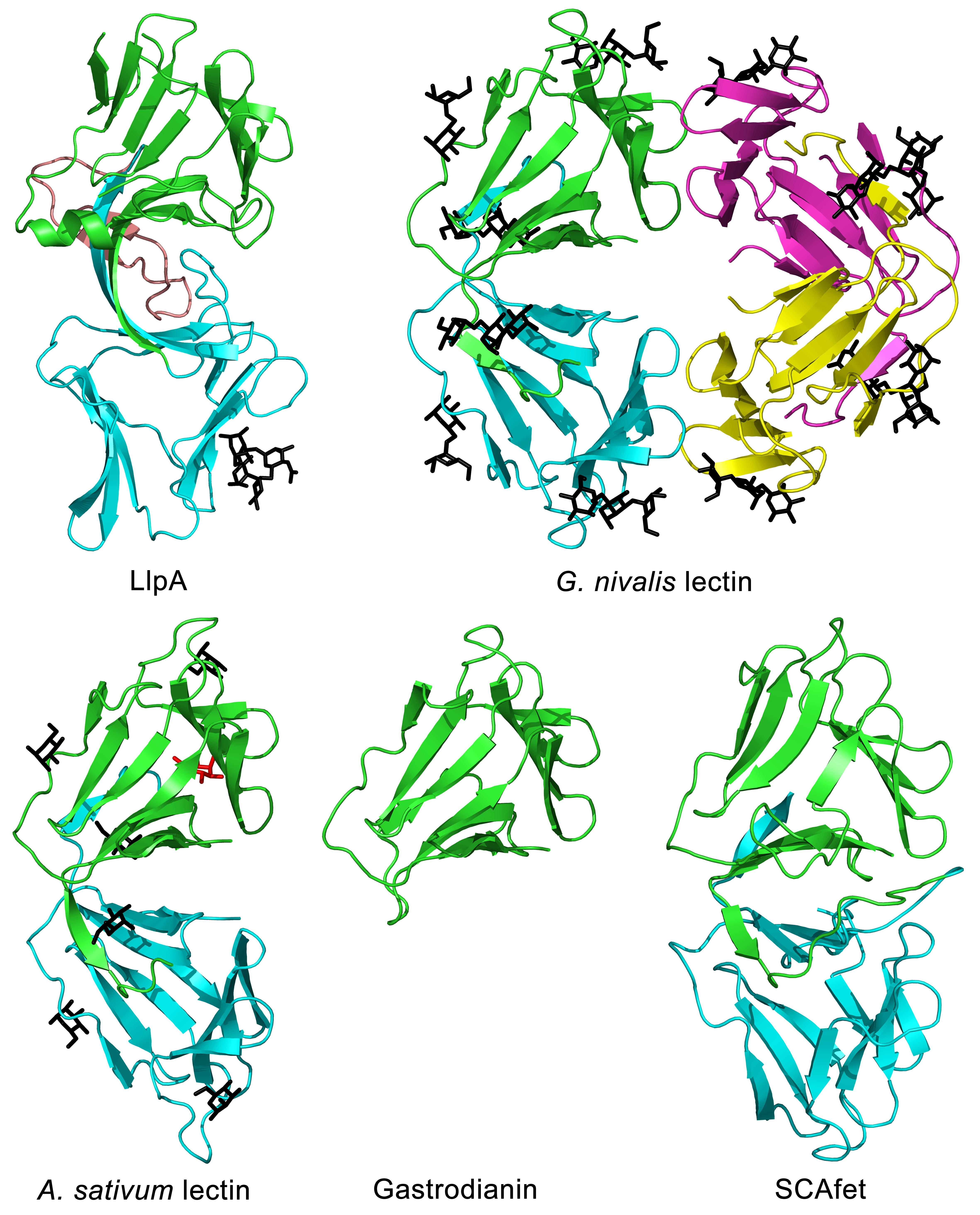

Supplement: Figure S2 — Quaternary structures and domain organization of various MMBL family members. Individual domains or protomers are shown in different colours. The domain or protomer colored green (which in the tandem MMBLs of LlpA, ASA I and SCAfet corresponds to the N-terminal domain) is always shown in the same orientation. Bound carbohydrates are shown in black stick representation. For LlpA a single pentasaccharide is bound to site IIIC. In the case of Galanthus nivalis (snowdrop) lectin (PDB entry 1JPC), twelve trimannosides are bound to all QxDxNxVxY motifs (three on each monomer of the homotetrameric protein). The snowdrop lectin tetramer consists of the association of two domain-swapped dimers (green-blue and pink-yellow). In the case of Allium sativum (garlic lectin ASA I - PDB entry 1KJ1), again each QxDxNxVxY motif has a dimannose bound while an additional sugar (shown in red) is bound to a non-canonical site. The protein is synthesized as a single chain precursor and post-translationally cleaved into two MMBL domains that adopt the same domain-swapped dimer as found in snowdrop lectin. Gastrodianin is a monomeric MMBL family member from the orchid Gastrodia elata (PDB entry 1XD5). The location(s) of its carbohydrate-binding site(s) is (are) not known. The fetuin-binding tandem-MMBL SCAfet from Scilla campanulata (PDB entry 1DLP) consists of two covalently attached MMBL domains, whereas in LlpA the swap of the C-terminal β-strands is retained. The relative orientation in the two domains is as in ASA I. This lectin binds fetuin rather than oligomannosides, but the locations of the binding sites are not known. (JPG) [file ppat.1003199.s002.jpg]

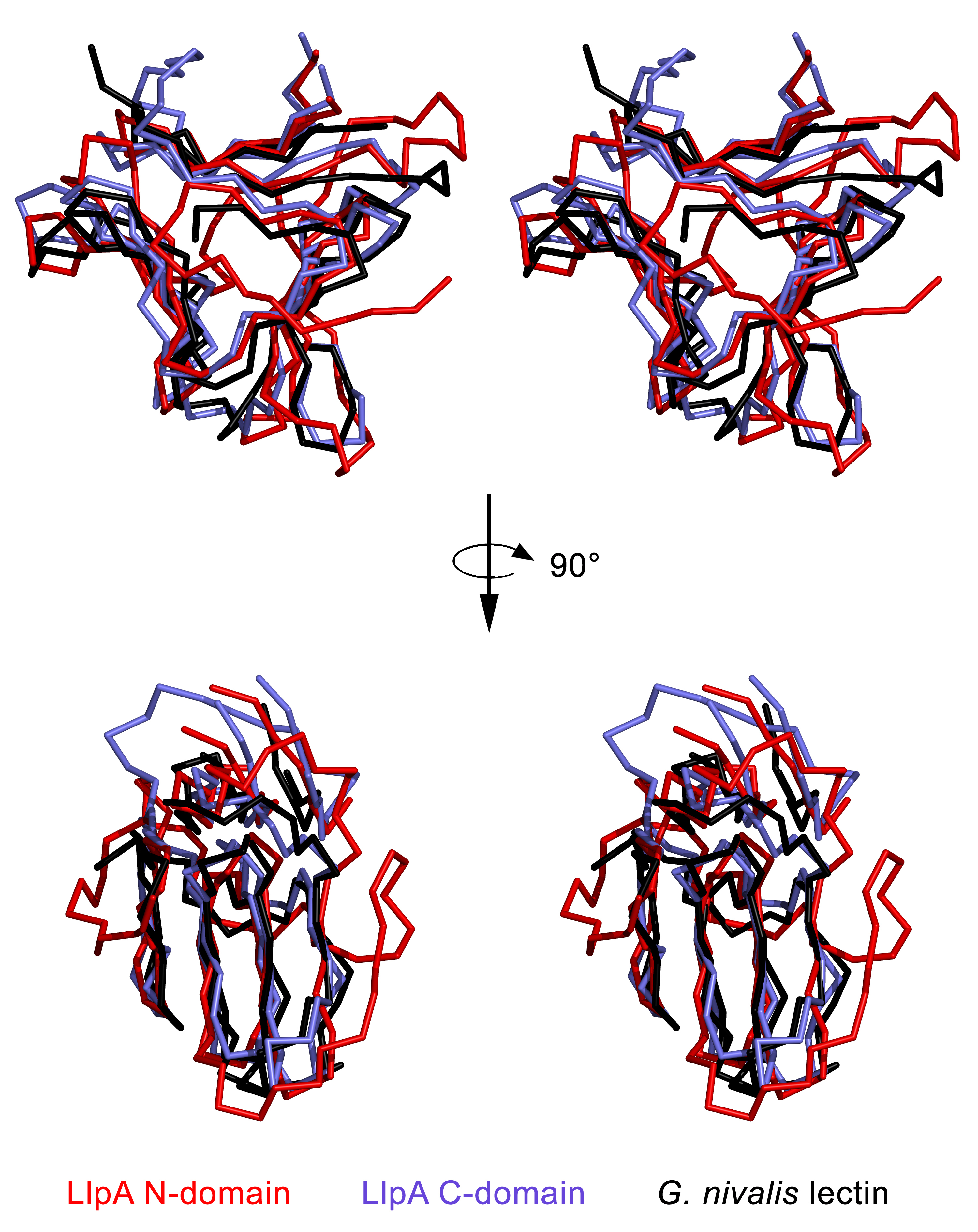

Supplement: Figure S3 — Stereo view of the superpositions (Cα representations) of the N-domain of LlpABW (red), C-domain of LlpABW (blue) and Galanthus nivalis lectin (PDB entry 1MSA, black). The superposition is shown in two orientations rotated by 90°. (JPG) [file ppat.1003199.s003.jpg]

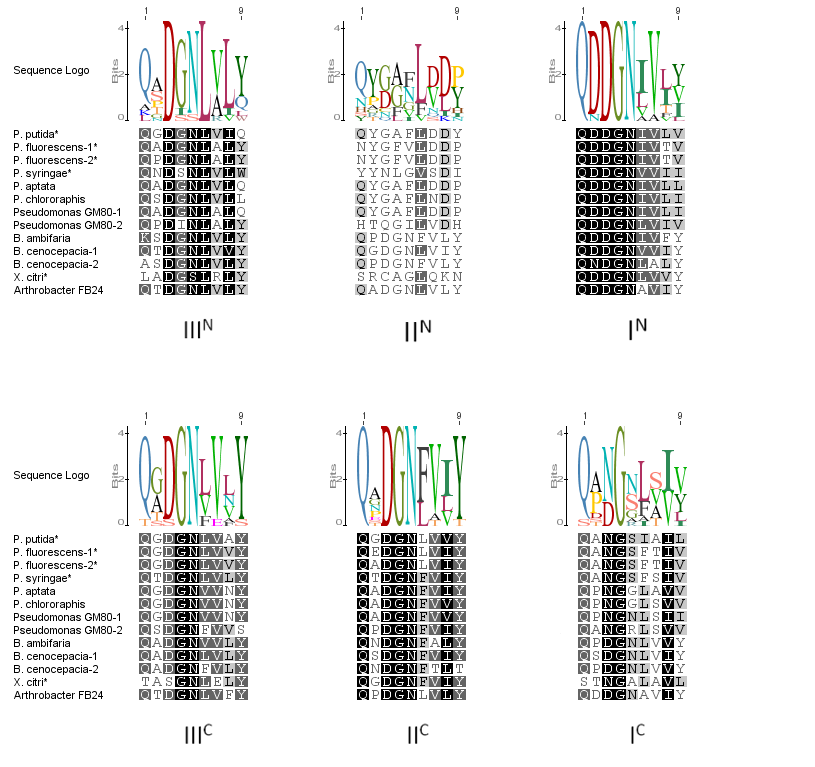

Supplement: Figure S4 — Sequence alignment of potential mannose-binding motifs in prokaryotic tandem MMBL proteins. The sequences corresponding to the consensus motif QxDxNxVxY, extracted from the N-domain and the C-domain of P. putida LlpABW and its homologues, are aligned per domain. Sequence conservation is visualized by differential shading. The sequence logo representation visualizes the degree of consensus for each residue. LlpA proteins with proven bacteriotoxic activity are labeled with an asterisk. Accession numbers: Arthrobacter sp. FB24 (YP_829274), Burkholderia ambifaria MEX-5 (ZP_02905572), Burkholderia cenocepacia AU 1054 ([1], ABF75998; [2], ABF75999), Pseudomonas chlororaphis subsp. aureofaciens 30–84 (EJL08681), Pseudomonas putida BW11M1 (AAM95702), Pseudomonas fluorescens Pf-5 (LlpA1 [1], YP_258360; LlpA2 [2], YP_259234), Pseudomonas sp. GM80 ([1], ZP_10606046; [2], ZP_10606131), Pseudomonas syringae pv. aptata DSM 50252 (EGH77666), Pseudomonas syringae pv. syringae 642 (ZP_07263221), Xanthomonas axonopodis pv. citri str. 306 (AAM35756). (TIF) [file ppat.1003199.s004.tif]

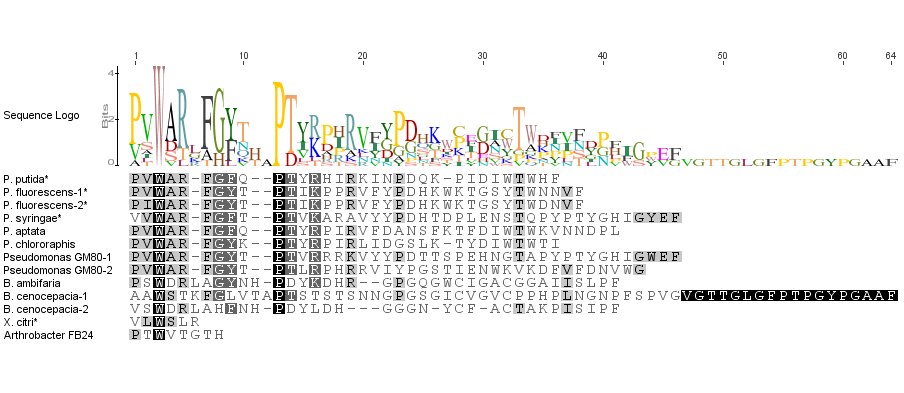

Supplement: Figure S5 — Sequence alignment of the carboxy-terminal sequences of LlpA-like proteins. The P. putida LlpABW sequence adopting a β-hairpin fold is delineated in Figure S1. The preceding conserved tryptophan residue is located C-terminally to IC (Figure S1). The sequence logo representation visualizes the degree of consensus for each residue. Accession numbers are listed in Figure S4. (TIF) [file ppat.1003199.s005.tif]

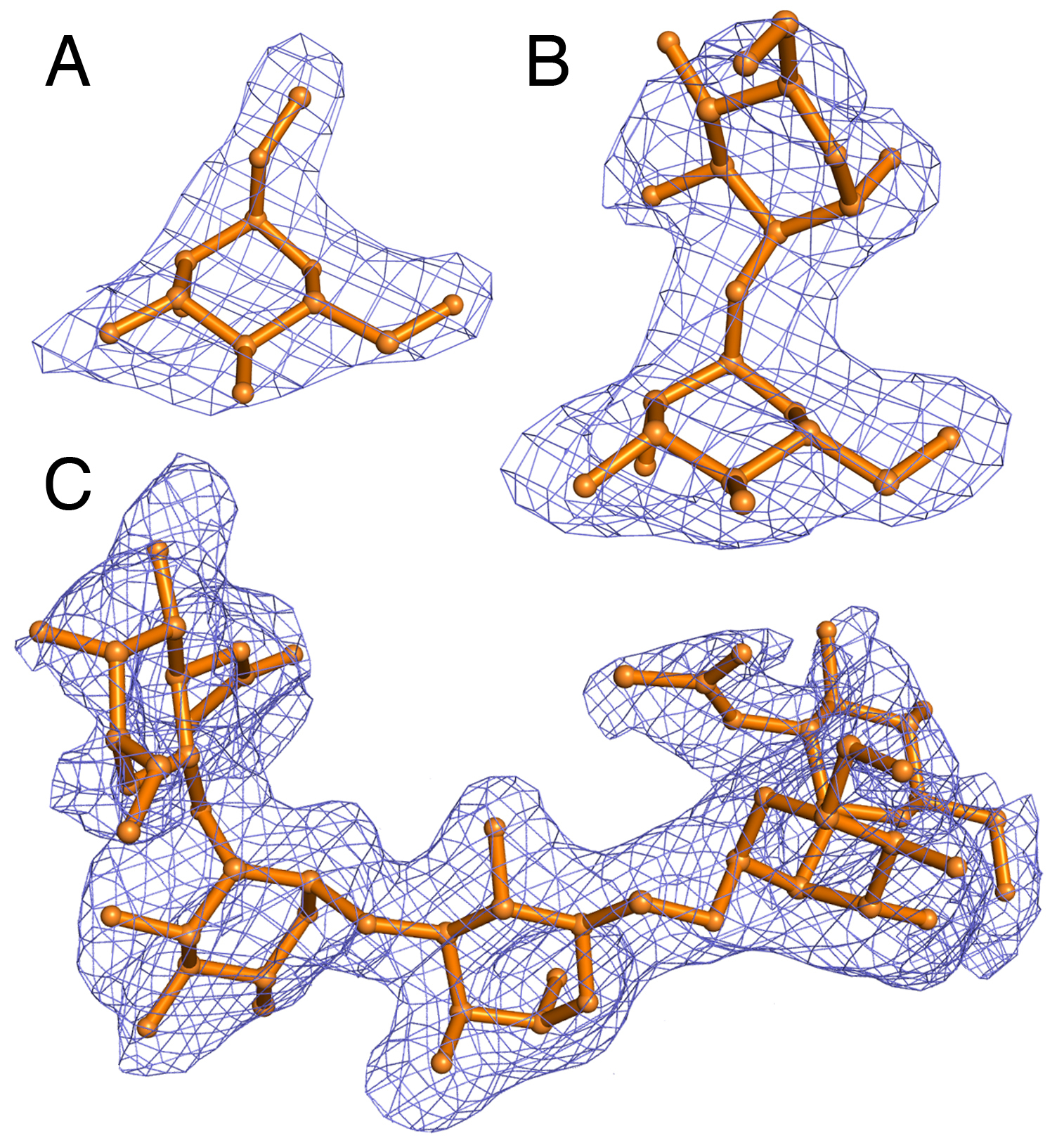

Supplement: Figure S6 — Electron density for (A) Methyl-α-D-Man, (B) Manα(1–2)Man and (C) GlcNAcβ(1–2)Manα(1–3)[GlcNAcβ(1–2)Manα(1–6)]Man. Difference electron-density maps are calculated by removing the sugar residues from the final coordinates and applying one round of slow-cool simulated annealing refinement to remove potential bias. The atomic model is superimposed in each case. (JPG) [file ppat.1003199.s006.jpg]

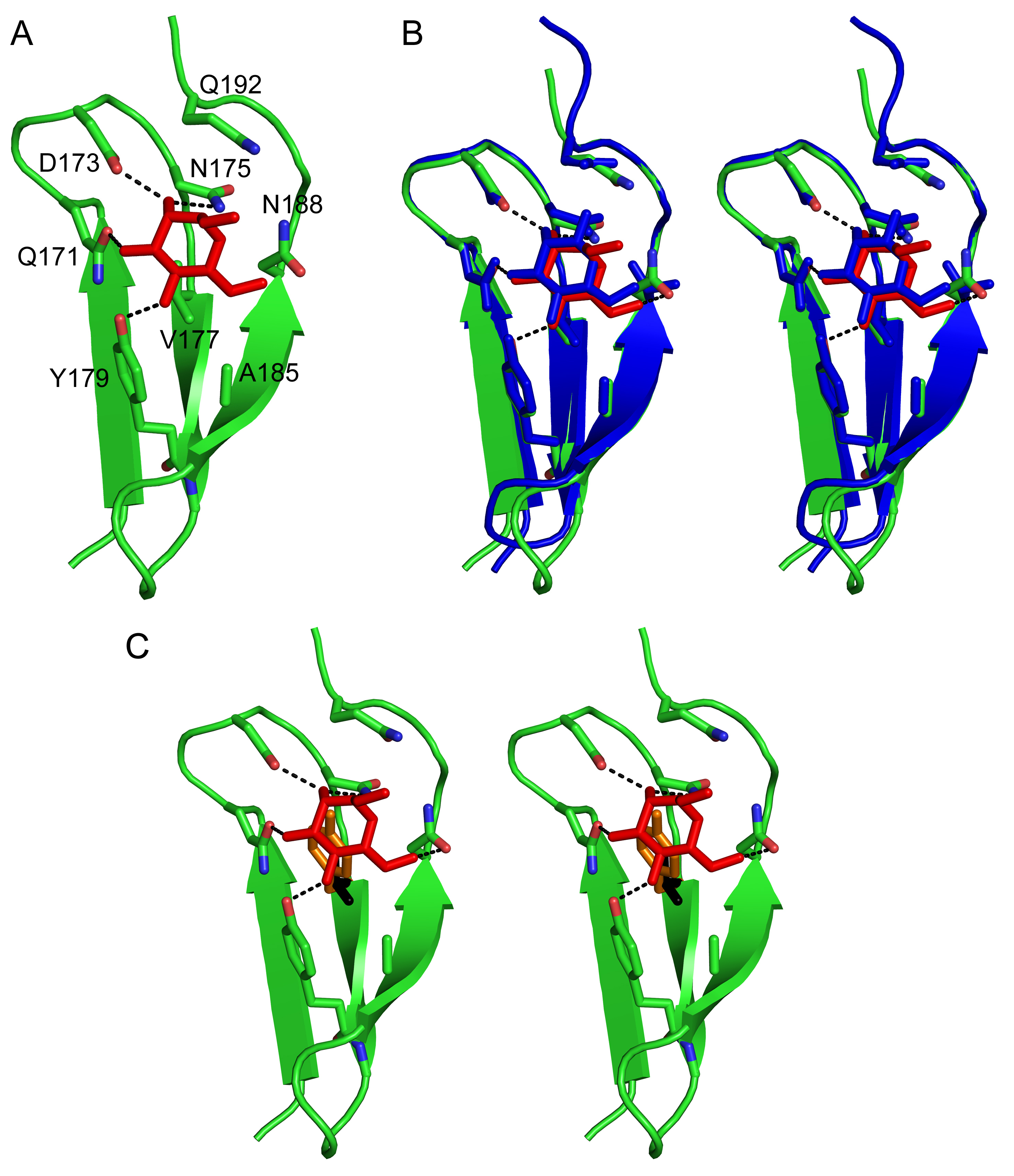

Supplement: Figure S7 — Mannose binding to LlpABW and garlic lectin. (A) Cartoon representation of subdomain IIIC of LlpABW (green) with residues implicated in carbohydrate binding showing in ball-and-stick representation and labeled (carbon green, oxygen red, nitrogen blue). The Me-Man residue is shown in red. Selected hydrogen bonds are shown as black dotted lines. (B) Stereoview of the superposition of subdomain IIIC of LlpABW (green) on the equivalent subdomain of garlic lectin (blue). The Me-Man residue bound to LlpABW is shown in red, the mannose bound to garlic lectin in blue. (C) Stereoview of the superposition of subdomain IIIC of LlpABW (green) identical as in panel A, but emphasizing the location of Val177 (shown as black sticks). The modeled Val177Tyr mutation is shown as orange sticks. Tyr177 makes a steric clash with the bound mannose (red) and is therefore expected to prevent binding, in agreement with our ITC experiments. (JPG) [file ppat.1003199.s007.jpg]

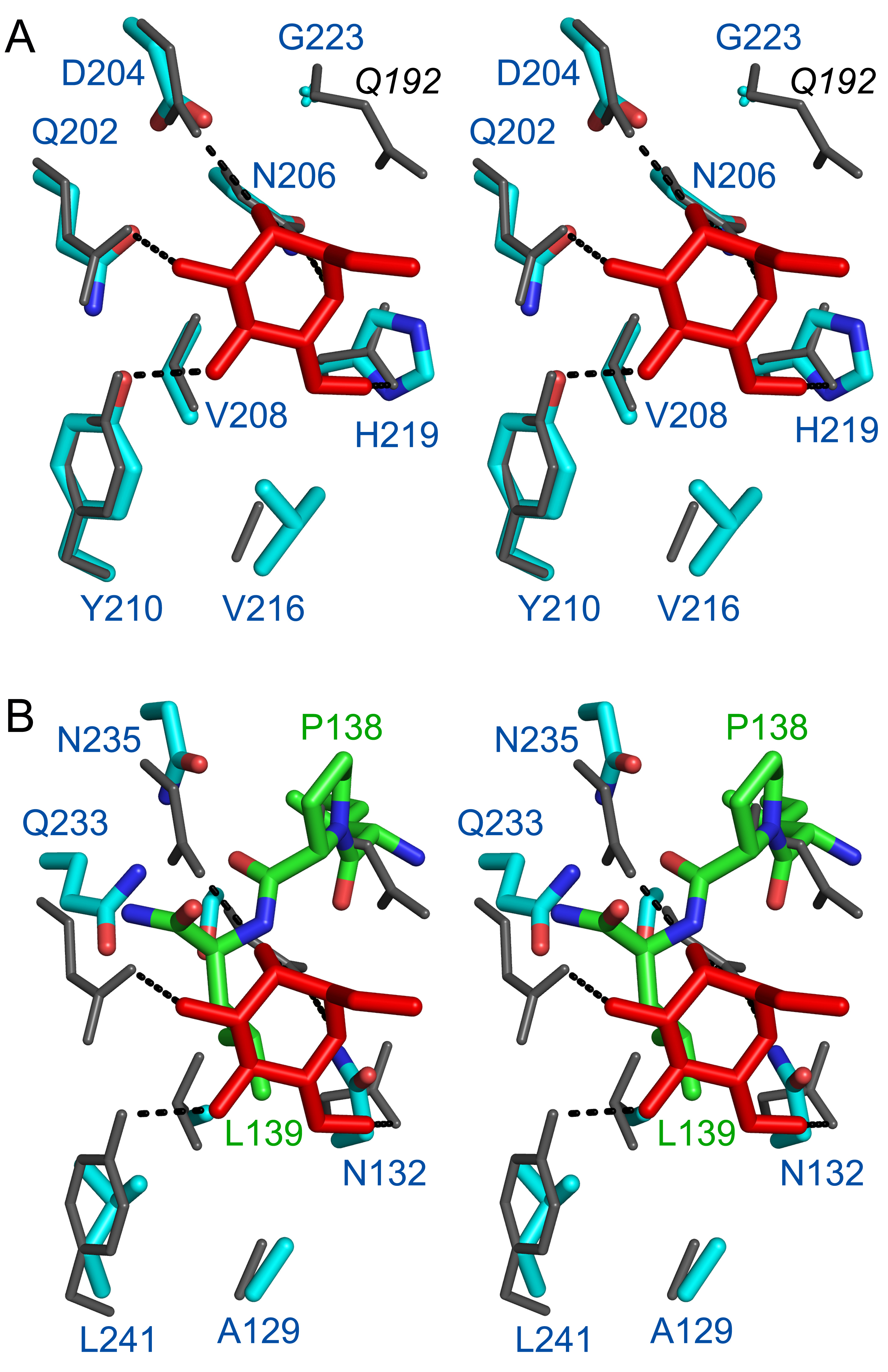

Supplement: Figure S8 — Sites II and I of the LlpABW C-domain. (A) Stereoview of site IIC of the C-domain (colored according to atom type) superimposed on site IIIC of the C-domain (dark gray). The Me-Man bound in site IIIC is shown in red. This site is very similar to site IIIC but in the crystal it is inaccessible due to crystal lattice interactions. Residue labels correspond to residues of site IIC. (B) Similar view showing site IC of the C-domain (colored according to atom type) superimposed on site IIIC of the C-domain (dark gray). The Me-Man bound in site IIIC is shown in red. The stretch of Ile137-Leu139 that provides a steric conflict preventing Me-Man binding in site IC, is highlighted with carbon atoms drawn in green. Residue labels correspond to residues of site IC. (JPG) [file ppat.1003199.s008.jpg]

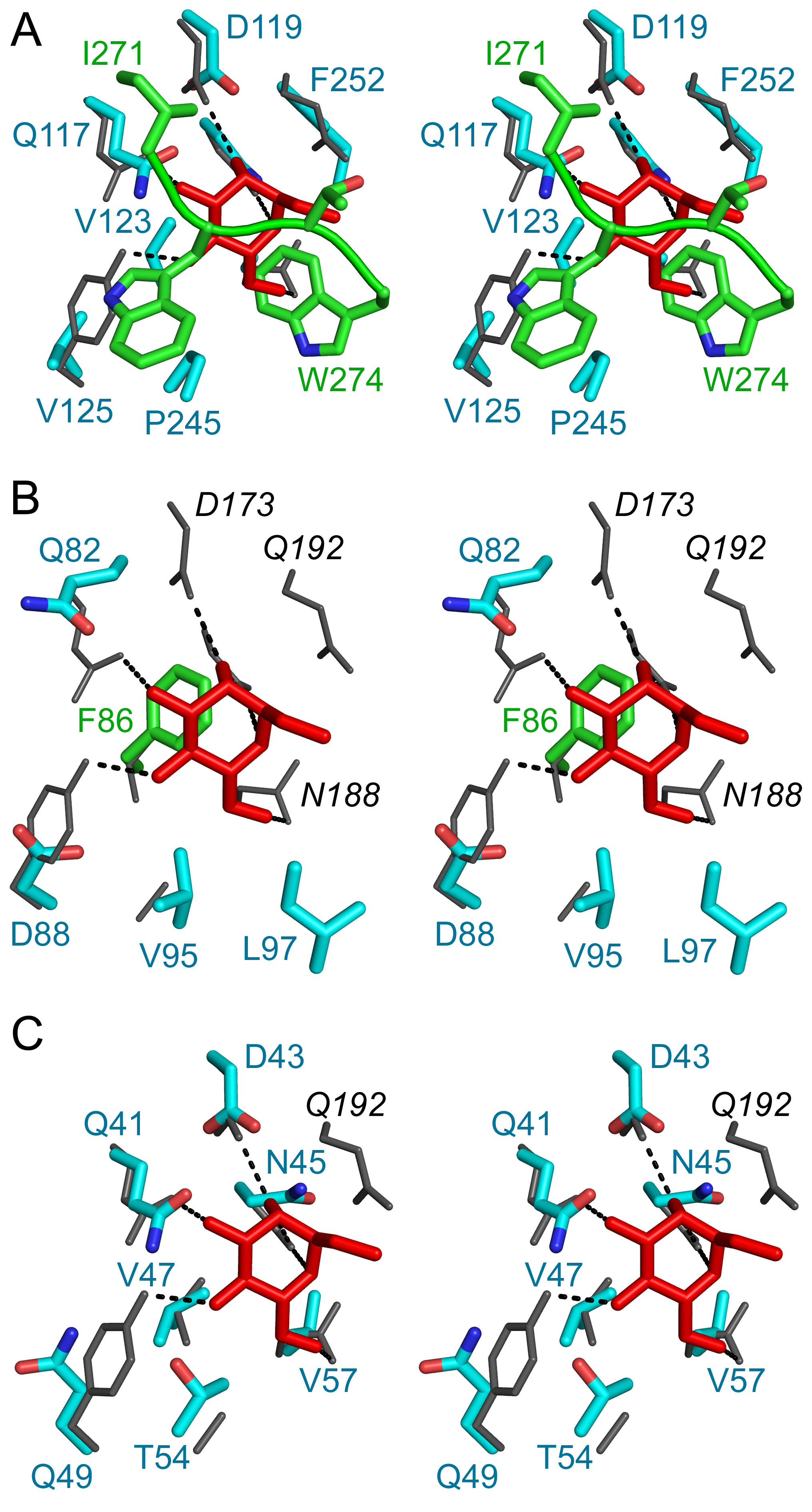

Supplement: Figure S9 — Sites of the LlpABW N-terminal domain. (A) Stereoview of site IN of the N-domain (colored according to atom type) superimposed on site IIIC of the C-domain (dark gray). The Me-Man bound in site IIIC is shown in red. The stretch of Ile271-Trp274 that provides a steric conflict preventing Me-Man binding in site IN is highlighted with carbon atoms drawn in green. Residue numbering corresponds to residues of site IN. (B) Similar superposition for site IIN of the N-domain. Phe86 that prevents Me-Man binding to this site through a steric conflict is highlighted in green. Other residues belonging to site IIN are labeled in teal. Three residues of site IIIC for which site IIN has no structural equivalent are labeled in black. (C) Similar superposition for site IIIN of the N-domain. Residues belonging to site IIIN are labeled in teal. One residue of site IIIC for which site IIIN has no structural equivalent, is labeled in black. For this site there are no obvious steric conflicts that would prevent positioning of a Me-Man residue although none is observed experimentally. (JPG) [file ppat.1003199.s009.jpg]

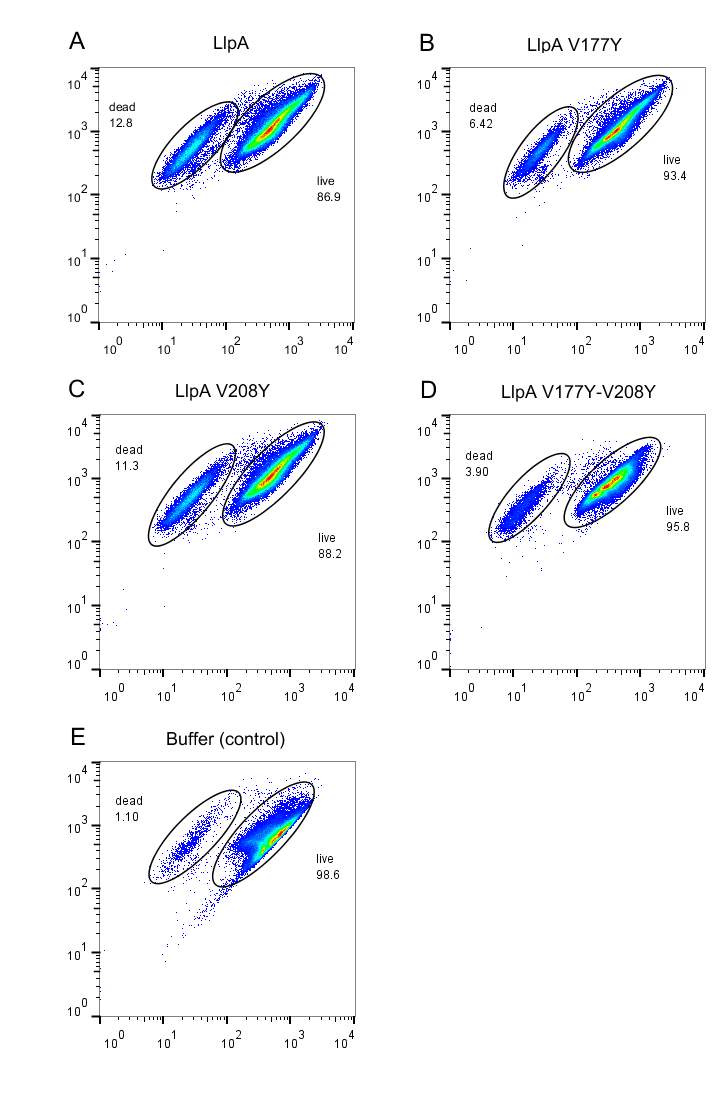

Supplement: Figure S10 — Quantification of live and dead cells by flow cytometry. P. syringae GR12-2R3 cells were treated with LlpA (A), LlpAV177Y (B), LlpAV208Y (C), LlpAV177Y-V208Y (D), or buffer (E) at a final concentration of 50 µg/ml for 1 h at 20°C. After live/dead staining, cells were analysed by flow cytometry. Data processing allowed to distinguish populations of dead (left) and live (right) cells. Spot densities ranging from high to low are differentiated by a color gradient from red, yellow, green, teal to blue. Representative samples for LlpA, mutant proteins and buffer control are shown in panels A–E. (TIF) [file ppat.1003199.s010.tif]

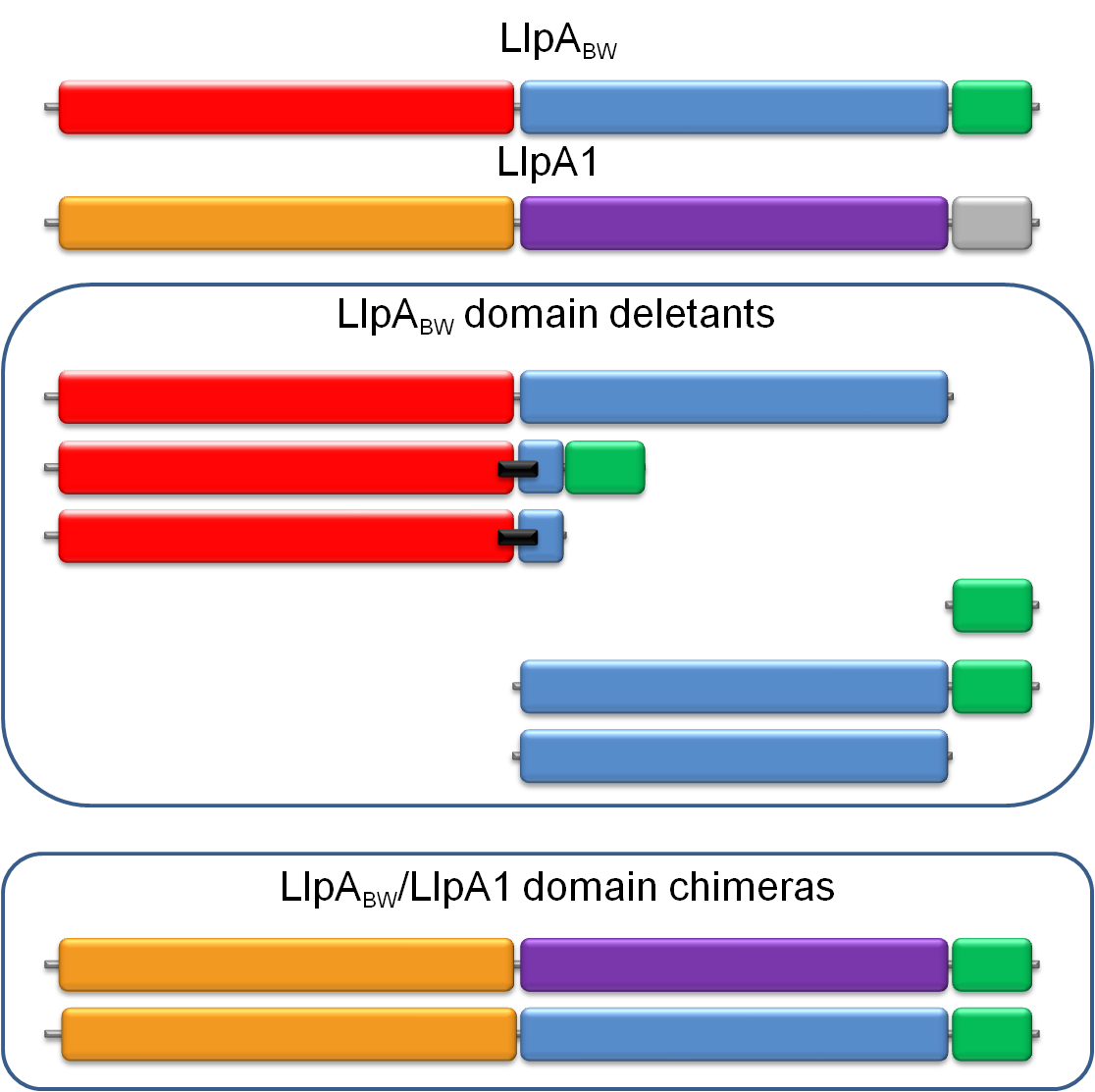

Supplement: Figure S11 — Overview of inactive LlpABW deletants and inactive LlpABW/LlpA1 chimers. The equivalent domains of LlpA1 are delineated based on pairwise sequence alignment with LlpABW: N-domain (orange), C-domain (purple), C-terminal extension (grey). No bacteriocin activity was conferred by these constructs upon recombinant E. coli cells tested against P. syringae GR12-2R3 (indicator strain for native LlpABW) and P. fluorescens LMG 1794 (indicator strain for native LlpA1). The small black rectangle represents an artificial linker sequence (DASRS). (TIF) [file ppat.1003199.s011.tif]
