# Supplementary material for: Structural Determinants for Activity and Specificity of the Bacterial Toxin LlpA
Source: PLoS Pathog. 2013 Feb 28;9(2):e1003199. doi: 10.1371/journal.ppat.1003199 (PMC3585409; doi:10.1371/journal.ppat.1003199)
Supplement: Table S2 — Bacterial strains and plasmids used in this study. (DOC) [file ppat.1003199.s013.doc]

# TABLE S2. Bacterial strains and plasmids used in this study.

| **Strain, plasmid, or primer** | | **Genotype, origin, or relevant characteristic^a^** | **Source or reference** |
| --- | --- | --- | --- |
| Bacteria | |  |  |
| *E. coli* strains | |  |  |
|  | TOP10F’ | F’[*lacI^q^* Tn*10* (Tc^r^)] *mcr*A Δ(*mrr-hsd*RMS*-mcr*BC) Φ80*lacZ*ΔM15 Δ*lac*X74  *rec*A1 *ara*D139 Δ(*ara-leu*)7697 *gal*U *gal*K *rps*L (Str^r^) *end*A1 *nup*G | Invitrogen |
|  | BL21(DE3) | F^–^ ompT hsdS_B_(r_B_^–^, m_B_^–^) gal dcm (DE3) | Novagen |
| *Pseudomonas* strains | |  |  |
|  | *P. syringae* GR12-2R3 | Rifampicin-resistant derivative of strain GR12-2, isolated from grasses (Canadian High Arctic) | [1,2] |
|  | *P. fluorescens* LMG 1794 | Type strain | BCCM^b^ |
|  |  |  |  |
| Plasmids | |  |  |
|  | pCMPG6053 | pUC18 with *llpA1* gene cloned in BamHI/SphI, Ap^r^ | [3] |
|  | pCMPG6056 | pET28 with N-terminal His_6_-tagged *llpA_BW_* gene cloned in NdeI/XhoI, Km^r^ | [4] |
|  | pCMPG6129 | pUC18 with 921-bp PCR-amplified fragment containing *llpA_BW_* gene cloned in KpnI/BamHI | This study |
|  | pCMPG6130 | pUC18 with 864-bp SOE-ligated PCR-amplified fragment containing domain deletant *llpA_BW_* gene (N-domain, C-domain), cloned in KpnI/BamHI | This study |
|  | pCMPG6131 | pUC18 with 537-bp SOE-ligated PCR-amplified containing domain deletant *llpA_BW_* gene (N-domain), cloned in KpnI/BamHI^c^ | This study |
|  | pCMPG6132 | pUC18 with 594-bp SOE-ligated PCR-amplified containing domain deletant *llpA_BW_* gene (N-domain, C-terminal extension), cloned in KpnI/BamHI^c^ | This study |
|  | pCMPG6133 | pUC18 with 918-bp SOE-ligated PCR-amplified fragment containing *llpA_BW_* gene lacking C-terminal Phe, cloned in KpnI/BamHI | This study |
|  | pCMPG6134 | pUC18 with 447-bp SOE-ligated PCR-amplified fragment containing *llpA_BW_* gene (C-domain), cloned in KpnI/BamHI | This study |
|  | pCMPG6135 | pUC18 with 546-bp SOE-ligated PCR-amplified fragment containing *llpA_BW_* gene (C-domain, C-terminal extension), cloned in KpnI/BamHI | This study |
|  | pCMPG6136 | pUC18 with 165-bp SOE-ligated PCR-amplified fragment containing *llpA_BW_* gene (C-terminal extension), cloned in *KpnI*/*BamHI* | This study |
|  | pCMPG6137 | pUC18 with 1059-bp SOE-ligated PCR-amplified fragment containing chimeric *llpA_BW_/llpA1* gene (N-domain*_llpABW_*,C-domain*_llpA1_*, C-term.ext.*_llpA1_*), cloned in BamHI/SphI | This study |
|  | pCMPG6138 | pUC18 with 972-bp SOE-ligated PCR-amplified fragment containing chimeric *llpA_BW_/llpA1* gene (N-domain*_llpA1_*,C-domain*_llpA1_*, C-term.ext.*_llpABW_*), cloned in BamHI/BamHI | This study |
|  | pCMPG6139 | pUC18 with 1110-bp SOE-ligated PCR-amplified fragment containing chimeric *llpA_BW_/llpA1* gene (N-domain*_llpA1_*,C-domain*_llpABW_*, C-term.ext.*_llpA1_*), cloned *in BamHI/SphI* | This study |
|  | pCMPG6140 | pUC18 with 1059-bp SOE-ligated PCR-amplified fragment containing chimeric *llpA_BW_/llpA1* gene (N-domain*_llpABW_*,C-domain*_llpABW_*, C-term.ext.*_llpA1_*), cloned in BamHI/SphI | This study |
|  | pCMPG6141 | pUC18 with 921-bp SOE-ligated PCR-amplified fragment containing chimeric *llpA_BW_/llpA1* gene (N-domain*_llpABW_*,C-domain*_llpA1_*, C-term.ext.*_llpABW_*), cloned in BamHI/BamHI | This study |
|  | pCMPG6142 | pUC18 with 972-bp SOE-ligated PCR-amplified fragment containing chimeric *llpA_BW_/llpA1* gene (N-domain*_llpA1_*,C-domain*_llpABW_*, C-term.ext.*_llpABW_*), cloned in BamHI/BamHI | This study |
|  | pCMPG6143 | pCMPG6129 with V47Y | This study |
|  | pCMPG6144 | pCMPG6129 with V177Y | This study |
|  | pCMPG6145 | pCMPG6129 with V208Y | This study |
|  | pCMPG6146 | pCMPG6129 with V47Y, V177Y | This study |
|  | pCMPG6147 | pCMPG6129 with V47Y, V208Y | This study |
|  | pCMPG6148 | pCMPG6129 with V177Y, V208Y | This study |
|  | pCMPG6149 | pCMPG6056 with V177Y | This study |
|  | pCMPG6150 | pCMPG6056 with V208Y | This study |
|  | pCMPG6151 | pCMPG6056 with V177Y, V208Y | This study |
|  | pCMPG6158 | pUC18 with 912-bp SOE-ligated PCR-amplified fragment containing *llpA_BW_* gene lacking C-terminal Trp-His-Phe, cloned in KpnI/BamHI | This study |
|  | pUC18/pUC19 | ColE1 replicon, *lacZ*, cloning vector; Ap^r^ | [5] |

^a^ Phenotypical resistance abbreviations: Ap, ampicillin; Km, kanamycin; Str, streptomycin; Tc, tetracycline

^b^ BCCM: Belgian Co-ordinated Collections of Micro-organisms (http://bccm.belspo.be/index.php/)

^c^ An artificial linker DASRS was used to connect S127 with D242 from LlpA_BW_

1. Lifshitz R, Kloepper JW, Kozlowski M, Simonson C, Carlson J, et al. (1987) Growth promotion of canola (rapeseed) seedlings by a strain of *Pseudomonas putida* under gnotobiotic conditions. Can J Microbiol 33: 390-395.
2. Blakney AJ, Patten CL (2011) A plant growth-promoting pseudomonad is closely related to the *Pseudomonas syringae* complex of plant pathogens. FEMS Microbiol Ecol 77: 546-557.
3. Parret AHA, Temmerman K, De Mot R (2005) Novel lectin-like bacteriocins of biocontrol strain *Pseudomonas fluorescens* Pf-5. Appl Environ Microbiol 71: 5197-5207.
4. Parret AHA, Wyns L, De Mot R, Loris R (2004) Overexpression, purification and crystallization of bacteriocin LlpA from *Pseudomonas putida* BW11M1. Acta Crystallogr D Biol Crystallogr 60: 1922-1924.
5. Yanisch-Perron C, Vieira J, Messing J (1985) Improved M13 phage cloning vectors and host strains: nucleotide sequences of the M13mp18 and pUC19 vectors. Gene 33: 103-119.
