# Supplementary material for: Structural Determinants for Activity and Specificity of the Bacterial Toxin LlpA
Source: PLoS Pathog. 2013 Feb 28;9(2):e1003199. doi: 10.1371/journal.ppat.1003199 (PMC3585409; doi:10.1371/journal.ppat.1003199)
Supplement: Table S3 — PCR primers used in this study. (DOCX) [file ppat.1003199.s014.docx]

**TABLE S3.** PCR primers used in this study.

| **Primer number** | **Primer sequence** | **Purpose of use** |
| --- | --- | --- |
| PGPRB-2545 | 5’-AAGTTGGGTAACGCCAGGGT-3’ | Reverse sequencing primer pUC18 |
| PGPRB-2546 | 5’-GCACCCCAGGCTTTACACTTT-3’ | Forward sequencing primer pUC18 |
| PGPRB-2810 | 5’- TGGCAGCAGCCAACTCAGCTT-3’ | Reverse sequencing primer pET28a |
| PGPRB-2811 | 5’- TATAGGCGCCAGCAACCGCA-3’ | Forward sequencing primer pET28a |
| PGPRB-3155 | 5’-TGGCTAGGTACCGCTCCCCTTCAATCGATCCAGTAGG-3’^a^ | Forward primer to construct pCMPG6129, pCMPG6130, pCMPG6131, pCMPG6132, pCMPG6133, pCMPG6134, pCMPG6135, pCMPG6136, pCMPG6140, pCMPG6158 |
| PGPRB-3156 | 5’-TGGCTAGGATCCAAGGCCGGGCCCGTTAAGGC-3’^a^ | Reverse primer to construct pCMPG6129, pCMPG6130, pCMPG6131, pCMPG6132, pCMPG6133, pCMPG6134, pCMPG6135, pCMPG6136, pCMPG6138, pCMPG6141, pCMPG6142, pCMPG6158 |
| PGPRB-4036 | 5’-TGGCTAGGATCCGCTCCCCTTCAATCGATCCAGTAGG-3’^a^ | Forward primer to construct pCMPG6137, pCMPG6141 |
| PGPRB-3151 | 5’- TGGCTAGGATCCCCTCGAGCCTCGAGCAATCG-3’^a^ | Forward primer to construct pCMPG6138, pCMPG6139, pCMPG6142 |
| PGPRB-3152 | 5’- TGGCTAGCATGCGGCAACTAAGCTGATGCGGC-3’^a^ | Reverse primer to construct pCMPG6137, pCMPG6139, pCMPG6140 |
| PGPRB-3868 | 5’-GCGATAGGTCGGCTGGAAGCCG-3’ | Reverse primer to construct pCMPG6130 |
| PGPRB-3869 | 5’-CGGCTTCCAGCCGACCTATCGCTGAGTCGGGCGCCGGGCCTTAACG-3’ | Forward primer to construct pCMPG6130 |
| PGPRB-2842 | 5’-CGAGCGCGACGCGTCCGAGTCGACCAGCACGATATT-3’ | Reverse primer to construct pCMPG6131, pCMPG6132 |
| PGPRB-2843 | 5’-GACTCGGACGCGTCGCGCTCGGACGAGAAACCGGTATGGGC-3’ | Forward primer to construct pCMPG6131, pCMPG6132 |
| PGPRB-3870 | 5’-GCGATAGGTCGGCTGGAAGCC-3’ | Reverse primer to construct pCMPG6131 |
| PGPRB-3871 | 5’-GGCTTCCAGCCGACCTATCGCTGAGTCGGGCGCCGGGCCTTAACG-3’ | Forward primer to construct pCMPG6131 |
| PGPRB-3872 | 5’-GTGCCAGGTCCAGATGTCGATCG-3’ | Reverse primer to construct pCMPG6133 |
| PGPRB-3873 | 5’-CCGATCGACATCTGGACCTGGCACTGAGTCGGGCGCCGGGCCTTAACG-3’ | Forward primer to construct pCMPG6133 |
| PGPRB-3874 | 5’-CATGGTGTTTCTCCTACTGGATCG-3’ | Reverse primer to construct pCMPG6134, pCMPG6135, pCMPG6136 |
| PGPRB-3875 | 5’-CGATCCAGTAGGAGAAACACCATGTCGCTGGCGCTGTGGAACGGCACGCC-3’ | Forward primer to construct pCMPG6134, pCMPG6135 |
| PGPRB-3876 | 5’-CTCGTCGAGGATGGCGATGCTGCCG-3’ | Reverse primer to construct pCMPG6134 |
| PGPRB-3877 | 5’-GGCAGCATCGCCATCCTCGACGAGTGAGTCGGGCGCCGGGCCTTAACG-3’ | Forward primer to construct pCMPG6134 |
| PGPRB-3878 | 5’-CGATCCAGTAGGAGAAACACCATGCCGACCTATCGCCATATCCGC-3’ | Forward primer to construct pCMPG6136 |
| PGPRB-3182 | 5’-CGCATACCAGTGAGGAATGGTGTCGACCAGCACGATATTGCC-3’ | Reverse primer to construct pCMPG6137, pCMPG6141 |
| PGPRB-3183 | 5’-ACCATTCCTCACTGGTATGCG-3’ | Forward primer to construct pCMPG6137, pCMPG6141 |
| PGPRB-3184 | 5’-GCCGTTCCACAGCGCCAGCGAGTCGACGGTTACGATGTTCCC-3’ | Reverse primer to construct pCMPG6139, pCMPG6142 |
| PGPRB-2864 | 5’-TCGCTGGCGCTGTGGAACGGC-3’ | Forward primer to construct pCMPG6139, pCMPG6142 |
| PGPRB-4333 | 5’-CCGACCTATCGCCATATCCGC-3’ | Forward primer to construct pCMPG6138, pCMPG6141 |
| PGPRB-4334 | 5’-CCAACCATCAAGCCTCCGCGT-3’ | Forward primer to construct pCMPG6139, pCMPG6140 |
| PGPRB-4336 | 5’-ACGCGGAGGCTTGATGGTTGGCTGGAAGCCGAAGCGCGCCCATAC-3’ | Reverse primer to construct pCMPG6139, pCMPG6140 |
| PGPRB-4338 | 5’-GCGGATATGGCGATAGGTCGGGGTGTACCCGAAACGAGCCCAGAC-3’ | Reverse primer to construct pCMPG6138, pCMPG6141 |
| PGPRB-4341 | 5’- GGCAATTTGTACATCCAGGACAACGGC-3’ | Forward primer to construct pCMPG6143, pCMPG6146, pCMPG6147 |
| PGPRB-4342 | 5’- GCCGTTGTCCTGGATGTACAAATTGCC-3’ | Reverse primer to construct pCMPG6143, pCMPG6146, pCMPG6147 |
| PGPRB-4343 | 5’- GGTAATCTCTACGCCTATGGCCCGAAC-3’ | Forward primer to construct pCMPG6144, pCMPG6146, pCMPG6148, pCMPG6149, pCMPG6151 |
| PGPRB-4344 | 5’- GTTCGGGCCATAGGCGTAGAGATTACC-3’ | Reverse primer to construct pCMPG6144, pCMPG6146, pCMPG6148, pCMPG6149, pCMPG6151 |
| PGPRB-4345 | 5’- GGCAACCTGTACGTCTACGGTGCCGGC-3’ | Forward primer to construct pCMPG6145, pCMPG6147, pCMPG6148, pCMPG6150, pCMPG6151 |
| PGPRB-4346 | 5’- GCCGGCACCGTAGACGTACAGGTTGCC-3’ | Reverse primer to construct pCMPG6145, pCMPG6147, pCMPG6148, pCMPG6150, pCMPG6151 |
| PGPRB-7255 | 5’-GGTCCAGATGTCGATCGGCTTC-3’ | Reverse primer to construct pCMPG6158 |
| PGPRB-7256 | 5’-AGAAGCCGATCGACATCTGGACCTGAGTCGGGCGCCGGGCCTTAACG-3’ | Forward primer to construct pCMPG6158 |

^a^ Restriction sites incorporated in primers are underlined: GGTACC, KpnI; GGATCC, BamHI; GCATGC, SphI.
