# Supplementary material for: Structural Determinants for Activity and Specificity of the Bacterial Toxin LlpA
Source: PLoS Pathog. 2013 Feb 28;9(2):e1003199. doi: 10.1371/journal.ppat.1003199 (PMC3585409; doi:10.1371/journal.ppat.1003199)
Supplement: Table S4 — Protein sequences of LlpABW deletants and LlpABW/LlpA1 chimers. (DOCX) [file ppat.1003199.s015.docx]

**Table S4. Protein sequences of LlpA_BW_ deletants and LlpA_BW_/LlpA1 chimers.**

| **Plasmid** | | **Protein sequence** |
| --- | --- | --- |
|  | **LlpA_BW_ deletants** |  |
|  | pCMPG6130 | MAGRTRIPFNGVGTSVLPAYQTLSAGQYLLSPNQRFKLLLQGDGNLVIQDNGATVWVANEQQPFSSTIPLRNKKAPLAFYVQYGAFLDDYSRRRVWLTDNSTFTSNDQWNRTHLVLQDDGNIVLVDSLALWNGTPAIPLVPGAIDSLLLAPGSELVQGVVYGAGASKLVFQGDGNLVAYGPNGAATWNAGTQGKGAVRAVFQGDGNLVVYGAGNAVLWHSHTGGHASAVLRLQANGSIAILDEKPVWARFGFQPTYR |
|  | pCMPG6131 | MAGRTRIPFNGVGTSVLPAYQTLSAGQYLLSPNQRFKLLLQGDGNLVIQDNGATVWVANEQQPFSSTIPLRNKKAPLAFYVQYGAFLDDYSRRRVWLTDNSTFTSNDQWNRTHLVLQDDGNIVLVDSDASRSDEKPVWARFGFQPTYR |
|  | pCMPG6132 | MAGRTRIPFNGVGTSVLPAYQTLSAGQYLLSPNQRFKLLLQGDGNLVIQDNGATVWVANEQQPFSSTIPLRNKKAPLAFYVQYGAFLDDYSRRRVWLTDNSTFTSNDQWNRTHLVLQDDGNIVLVDSDASRSDEKPVWARFGFQPTYRHIRKINPDQKPIDIWTWHF |
|  | pCMPG6133 | MAGRTRIPFNGVGTSVLPAYQTLSAGQYLLSPNQRFKLLLQGDGNLVIQDNGATVWVANEQQPFSSTIPLRNKKAPLAFYVQYGAFLDDYSRRRVWLTDNSTFTSNDQWNRTHLVLQDDGNIVLVDSLALWNGTPAIPLVPGAIDSLLLAPGSELVQGVVYGAGASKLVFQGDGNLVAYGPNGAATWNAGTQGKGAVRAVFQGDGNLVVYGAGNAVLWHSHTGGHASAVLRLQANGSIAILDEKPVWARFGFQPTYRHIRKINPDQKPIDIWTWH |
|  | pCMPG6134 | MSLALWNGTPAIPLVPGAIDSLLLAPGSELVQGVVYGAGASKLVFQGDGNLVAYGPNGAATWNAGTQGKGAVRAVFQGDGNLVVYGAGNAVLWHSHTGGHASAVLRLQANGSIAILDE |
|  | pCMPG6135 | MSLALWNGTPAIPLVPGAIDSLLLAPGSELVQGVVYGAGASKLVFQGDGNLVAYGPNGAATWNAGTQGKGAVRAVFQGDGNLVVYGAGNAVLWHSHTGGHASAVLRLQANGSIAILDEKPVWARFGFQPTYRHIRKINPDQKPIDIWTWHF |
|  | pCMPG6136 | MPTYRHIRKINPDQKPIDIWTWHF |
|  | pCMPG6158 | MAGRTRIPFNGVGTSVLPAYQTLSAGQYLLSPNQRFKLLLQGDGNLVIQDNGATVWVANEQQPFSSTIPLRNKKAPLAFYVQYGAFLDDYSRRRVWLTDNSTFTSNDQWNRTHLVLQDDGNIVLVDSLALWNGTPAIPLVPGAIDSLLLAPGSELVQGVVYGAGASKLVFQGDGNLVAYGPNGAATWNAGTQGKGAVRAVFQGDGNLVVYGAGNAVLWHSHTGGHASAVLRLQANGSIAILDEKPVWARFGFQPTYRHIRKINPDQKPIDIWT |
|  | **LlpA_BW_/LlpA1 chimers** |  |
|  | pCMPG6137 | MAGRTRIPFNGVGTSVLPAYQTLSAGQYLLSPNQRFKLLLQGDGNLVIQDNGATVWVANEQQPFSSTIPLRNKKAPLAFYVQYGAFLDDYSRRRVWLTDNSTFTSNDQWNRTHLVLQDDGNIVLVDTIPHWYAPNGRVFTPAVGAAMIIGGTTELVPGQFYSAGEHSLVFQGDGNLVVYGPNSSVIWATYTQNKGGVRCVMQEDGNLVIYAANNGVVWQSGTGGHPGATIRLQANGSFTIVTERPVWARFGYTPTIKPPRVFYPDHKWKTGSYTWNNVF |
|  | pCMPG6138 | MAWIRYDFTDNGSSVLPARYYMAPNQYLQSPNKRFKLLFQADGNLALYDGAQLAWVADQNTPFTNVSKGNKKDPMMVFMNYGFVLDDPLRGRIWSTTPSDPTMGSREDASLRAFTQVQDDGNIVTVDTIPHWYAPNGRVFTPAVGAAMIIGGTTELVPGQFYSAGEHSLVFQGDGNLVVYGPNSSVIWATYTQNKGGVRCVMQEDGNLVIYAANNGVVWQSGTGGHPGATIRLQANGSFTIVTERPVWARFGYTPTYRHIRKINPDQKPIDIWTWHF |
|  | pCMPG6139 | MAWIRYDFTDNGSSVLPARYYMAPNQYLQSPNKRFKLLFQADGNLALYDGAQLAWVADQNTPFTNVSKGNKKDPMMVFMNYGFVLDDPLRGRIWSTTPSDPTMGSREDASLRAFTQVQDDGNIVTVDSLALWNGTPAIPLVPGAIDSLLLAPGSELVQGVVYGAGASKLVFQGDGNLVAYGPNGAATWNAGTQGKGAVRAVFQGDGNLVVYGAGNAVLWHSHTGGHASAVLRLQANGSIAILDEKPVWARFGFQPTIKPPRVFYPDHKWKTGSYTWNNVF |
|  | pCMPG6140 | MAGRTRIPFNGVGTSVLPAYQTLSAGQYLLSPNQRFKLLLQGDGNLVIQDNGATVWVANEQQPFSSTIPLRNKKAPLAFYVQYGAFLDDYSRRRVWLTDNSTFTSNDQWNRTHLVLQDDGNIVLVDSLALWNGTPAIPLVPGAIDSLLLAPGSELVQGVVYGAGASKLVFQGDGNLVAYGPNGAATWNAGTQGKGAVRAVFQGDGNLVVYGAGNAVLWHSHTGGHASAVLRLQANGSIAILDEKPVWARFGFQPTIKPPRVFYPDHKWKTGSYTWNNVF |
|  | pCMPG6141 | MAGRTRIPFNGVGTSVLPAYQTLSAGQYLLSPNQRFKLLLQGDGNLVIQDNGATVWVANEQQPFSSTIPLRNKKAPLAFYVQYGAFLDDYSRRRVWLTDNSTFTSNDQWNRTHLVLQDDGNIVLVDTIPHWYAPNGRVFTPAVGAAMIIGGTTELVPGQFYSAGEHSLVFQGDGNLVVYGPNSSVIWATYTQNKGGVRCVMQEDGNLVIYAANNGVVWQSGTGGHPGATIRLQANGSFTIVTERPVWARFGYTPTYRHIRKINPDQKPIDIWTWHF |
|  | pCMPG6142 | MAWIRYDFTDNGSSVLPARYYVAPNQYLQSPNKRFKLLFQADGNLALYDGAQLAWVADQNTPFTNVSKGNKKDPMMVFMNYGFVLDDPLRGRIWSTTPSDPTMGSREDASLRAFTQVQDDGNIVTVDSLALWNGTPAIPLVPGAIDSLLLAPGSELVQGVVYGAGASKLVFQGDGNLVAYGPNGAATWNAGTQGKGAVRAVFQGDGNLVVYGAGNAVLWHSHTGGHASAVLRLQANGSIAILDEKPVWARFGFQPTYRHIRKINPDQKPIDIWTWHF |
