# Supplementary material for: Structural Determinants for Activity and Specificity of the Bacterial Toxin LlpA
Source: PLoS Pathog. 2013 Feb 28;9(2):e1003199. doi: 10.1371/journal.ppat.1003199 (PMC3585409; doi:10.1371/journal.ppat.1003199)
Supplement: Table S5 — Structure determination and refinement. (DOCX) [file ppat.1003199.s016.docx]

**Table S5. Structure determination and refinement.**

|  |  | **Native** | **PCMB derivative** | **LlpA - Me-α-D-Man complex** | **LlpA - Manα(1-2)Man complex** | **LlpA - GlcNAcβ(1-2)Manα(1-3)[GlcNAcβ(1-2)Manα(1-6)]Man complex** |
| --- | --- | --- | --- | --- | --- | --- |
| **Data collection** |  |  |  |  |  |  |
| Wavelength |  | 0.8073 | 0.8073 | 0.8073 | 0.8081 | 0.8081 |
| Space group |  | P2_1_2_1_2 | P2_1_2_1_2 | P2_1_2_1_2 | P2_1_2_1_2 | P2_1_2_1_2 |
| Unit cell | a (Å)  b (Å)  c (Å) | 150.5  154.5  34.2 | 148.6  153.1  33.9 | 149.0  153.2  33.9 | 149.5  153.1  34.0 | 149.2  153.0  34.0 |
| Resolution range |  | 20.0-2.20 (2.28-2.20) | 50.0-3.60 (3.33-3.60) | 19.6-2.26 (2.34-2.26) | 19.7-2.08 (2.15-2.08) | 19.9-2.12 (2.17-2.12) |
| Total No. reflections measured |  | 210976 | 57398 | 212740 | 360137 | 288093 |
| No. unique reflections |  | 41385 (4097) | 9577 (966) | 33688 (3360) | 48079 (4731) | 45329 (4392) |
| Data completeness (%) |  | 99.9 (99.7) | 99.5 (100.0) | 90.0 (93.1) | 99.91 (100.00) | 99.96 (100.00) |
| Total oscillation range covered (°) |  | 130 | 150 | 140 | 175 | 140 |
| R_sym_ (%) |  | 6.4 (58.2) | 8.4 (14.5) | 12.4 (54.2) | 7.6 (40.2) | 8.1 (63.3) |
| I/Sig(I) |  | 16.2 (3.3) | 25.0 (14.1) | 7.1 (2.6) | 13.37 (2.84) | 12.18 (2.51) |
|  |  |  |  |  |  |  |
| **Phasing** |  |  |  |  |  |  |
| R_iso_ (%) |  | - | 20.7 | - |  |  |
| <d’’/sig> at resolution cutoff |  | - | 0.94 | - |  |  |
| FOM |  | - | 0.15 | - |  |  |
| Phasing Power |  |  | 0.73 |  |  |  |
| **Refinement** |  |  |  |  |  |  |
| R-factor |  | 0.177 | - | 0.175 | 0.158 | 0.155 |
| R_free_-factor |  | 0.204 | - | 0.212 | 0.200 | 0.188 |
| Number of atoms |  | 4192 | - | 4169 | 4437 | 4416 |
| B-factors (Å^2^) | From Wilson plot  All atoms  Protein atoms  Ligand atoms  Water atoms | 35.7  44.2  44.5  -  43.6 | -  -  -  -  - | 34.3  45.2  43.1  53.6  40.8 | 36.2  41.0  40.6  53.9  46.1 | 35.3  42.6  42.1  62.8  46.2 |
| R.m.s. deviations | Bond lengths (Å)  Bond angles (°) | 0.006  1.05 | -  - | 0.006  1.108 | 0.009  1.24 | 0.009  1.14 |
| Ramachandran profile | Core  Other allowed  Disallowed | 95.5  4.2  0.3 | -  -  - | 96.5  3.2  0.3 | 97.0  2.6  0.4 | 97.0  2.8  0.2 |
| PDB entries |  | 3M7H | - | 3M7J | 4GC1 | 4GC2 |
